# The Emergence and Evolution of Borophene

**DOI:** 10.1002/advs.202001801

**Published:** 2021-05-02

**Authors:** Meitong Ou, Xuan Wang, Liu Yu, Chuang Liu, Wei Tao, Xiaoyuan Ji, Lin Mei

**Affiliations:** ^1^ School of Pharmaceutical Sciences (Shenzhen) Sun Yat‐sen University Guangzhou 510275 P. R. China; ^2^ Center for Nanomedicine and Department of Anesthesiology Brigham and Women's Hospital Harvard Medical School Boston MA 02115 USA; ^3^ Academy of Medical Engineering and Translational Medicine Tianjin University Tianjin 300072 China; ^4^ Institute of Biomedical Engineering Chinese Academy of Medical Sciences and Peking Union Medical College Tianjin 300192 China

**Keywords:** biomedical applications, borophene, graphene analogues, synthesis, 2D nanomaterials

## Abstract

Neighboring carbon and sandwiched between non‐metals and metals in the periodic table of the elements, boron is one of the most chemically and physically versatile elements, and can be manipulated to form dimensionally low planar structures (borophene) with intriguing properties. Herein, the theoretical research and experimental developments in the synthesis of borophene, as well as its excellent properties and application in many fields, are reviewed. The decade‐long effort toward understanding the size‐dependent structures of boron clusters and the theory‐directed synthesis of borophene, including bottom‐up approaches based on different foundations, as well as up‐down approaches with different exfoliation modes, and the key factors influencing the synthetic effects, are comprehensively summarized. Owing to its excellent chemical, electronic, mechanical, and thermal properties, borophene has shown great promise in supercapacitor, battery, hydrogen‐storage, and biomedical applications. Furthermore, borophene nanoplatforms used in various biomedical applications, such as bioimaging, drug delivery, and photonic therapy, are highlighted. Finally, research progress, challenges, and perspectives for the future development of borophene in large‐scale production and other prospective applications are discussed.

## Graphical abstract

The successful theoretical‐research‐guided synthesis of borophene marks a milestone for the synthesis of 2D materials without layered bulk structures. The excellent properties and wide applications of borophene make it a new cousin of graphene in the family of 2D materials.

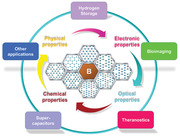

## 1 Introduction

2D nanomaterials have attracted major attention from the scientific community for their unique and superlative properties.^[^ ^1^, ^2^ ^]^ Compared with bulk materials, ultrathin 2D nanosheets with the majority of atoms exposed to the surface have greater surface areas, higher 2D nanosheet chemical and physical activity, and quantum confinement effects that endow them with special photonic, electronic, catalytic, and magnetic properties, and have great application potential in bio‐like materials, drug carriers, biosensors, electronic devices, etc.^[^ ^3^, ^4^, ^5^, ^6^ ^]^ In 2004, the emergence of graphene led to a great response in the material field and greatly improved the applications of 2D materials in various fields.^[^ ^7^, ^8^ ^]^ However, the zero bandgap of graphene hinders many applications of graphene in electronic components, biological imaging, and photodynamic therapy. In recent years, researchers have been trying to find 2D materials with honeycomb structure like graphene, or monoelemental 2D nanosheets that are in the same group as or adjacent to carbon element, hoping to develop graphene‐like materials with excellent properties. Fortunately, the emergence of the graphene‐like structure materials represented by transition metal sulfides (TMDs),^[^ ^9^, ^10^ ^]^ square boron nitride (h‐BN),^[^ ^11^ ^]^ and graphite phase carbon nitride (g‐C_3_N_4_)^[^ ^12^ ^]^ as well as monoelemental 2D materials represented by borophene,^[^ ^13^ ^]^ silicene,^[^ ^14^, ^15^, ^16^ ^]^ stanene,^[^ ^17^, ^18^ ^]^ and germanene^[^ ^19^ ^]^ can not only make up for the shortcoming of the graphene of zero bandgap, but they also have some more special properties, which are expected to bring new functions and applications. Compared with other 2D materials, monoelemental nanomaterials have three unique advantages: I) More compatible with existing semiconductor technology. For example, the silicon and germanium elements are the main elements to construct traditional semiconductor materials. II) Relatively simple to be synthesized with high quality due to being composed of one element. III) Easier to be degraded and metabolized by biological systems. Black phosphorus is one of the monoelemental 2D nanomaterials with good biocompatibility. It can be degraded into phosphate in vivo, and participate in maintaining many important physiological activities as a raw material for ATP and DNA.^[^ ^20^, ^21^ ^]^ In addition, the ultrahigh specific surface area and different levels of response to pH, light, electricity, etc., make the monoelemental 2D materials become superior candidates for electron devices, drug delivery, optical therapy, biological imaging, and other fields.^[^ ^22^, ^23^, ^24^ ^]^ As one of the monoelemental 2D materials, borophene is analogous to graphene and black phosphorus, not only possessing high specific surface area and drug loading capacity (borophene: 114%,^[^ ^25^ ^]^ black phosphorus: 108%,^[^ ^26^ ^]^ graphene oxide: 200%,^[^ ^27^ ^]^ other nanomaterials: 10–30%), but also responsively releasing drugs under pH, optical, and thermal stimulation. Borophene is one of the most mysterious monoelemental 2D nanomaterials, whose polymorphism is the most unique characteristic compared with other monoelemental 2D materials. Borophenes synthesized by different methods and conditions have different structures, and these allotropes possess different properties. For instance, *Pmmn* borophene is predicted to be the intriguing Dirac materials with Dirac cones and novel electrical characteristics;^[^ ^28^ ^]^ The properties of *β*_12_ phase borophene and *α* phase borophene are different; the former is highly anisotropic while the latter is isotropic;^[^ ^29^, ^30^ ^]^ This means that we can regulate and control the preparation conditions to synthesize borophene to meet application requirements. Nowadays, there are still few reports about borophene, and these intriguing properties that have been observed are just a small part of the iceberg, there are still many details for researchers to further exploit.

Boron is the only semiconductor element in Group IIIA, the fifth element in the periodic table of the elements, adjacent to and with valence orbitals similar to carbon.^[^ ^31^ ^]^ These similarities imply that borophene, the lighted 2D metal, may have interesting characteristics similar to those of graphene.^[^ ^32^, ^33^ ^]^ The special electronic structure, the complex bonding mechanism, and the close correlation with carbon elements indicate that borophene will have excellent properties mirroring those of graphene, and even surpass graphene as a new supermaterial. Indeed, theoretical calculations have implied that borophene has many low‐dimensional allotropes and interesting properties.^[^ ^34^ ^]^ Boustani et al.^[^ ^35^ ^]^predicted that boron clusters may form a quasi‐planar structure, indicating that 2D borophene can be prepared experimentally. The experimental synthesis of borophene was not achieved until Guisinger's^[^ ^36^ ^]^ research group and Wu's^[^ ^13^ ^]^ research group separately synthesized borophene on Ag(111) in 2015, despite boron's complex bonding mechanisms and non‐layered structure of bulk boron.

**Scheme 1:**
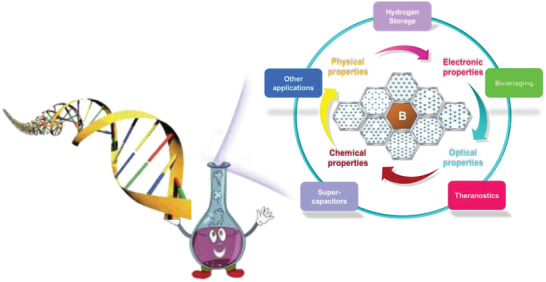
Illustration of the unique development of borophene. The theories of borophene are abundant. Key information affecting the properties and synthesis of borophene has been obtained through theoretical prediction, similar to gene‐directed protein synthesis, to generally guide the synthesis of borophene.

Unlike most traditional materials designed by direct experimental testing, the design of borophene begins with theories predicting the potential characteristics and possible preparation methods, successfully guiding the laboratory preparation and further application of borophene (**Scheme** **1**). The development of borophene is undoubtedly a successful example of the material genome project (MGI). Similar to human genes, materials also have key “genes” that determine their properties. MGI advocates that, prior to exploring unknown materials, the relationship between material composition, structure, and property should be clarified through theoretical research, so as to improve the efficiency of material synthesis, reduce the cost of material research, and increase the success rate of material design. The emergence and evolution of borophene provides an outstanding model for the synthesis of new materials.

In recent years, borophene, as a new supermaterial, has attracted extensive attention from scientific. The citation rate of borophene‐related papers has shown a rapid upward trend in the past 5 years. However, comprehensive review papers on borophene are rare. Ref. [37] elaborates on the dominant structure of borophene formation under different conditions; ref. [38] provides a good summary of how theory has guided the experimental synthesis of borophene and systematically analyzes the future application prospects of borophene. Ref. [39] is a good summary of the properties of borophene. The above reviews provide a good summary of the relationship between the structure and properties of borophene, without addressing the synthetic methods and applications of this interesting material. The present review will focus on the synthetic methods, properties, and applications of borophene, particularly in the field of biomedicine. We hope to construct an information blueprint of borophene to help readers obtain important information for the material's synthesis and applications.

## 2 Theoretical Study on the Structure of 2D Boron

**Figure 1:**
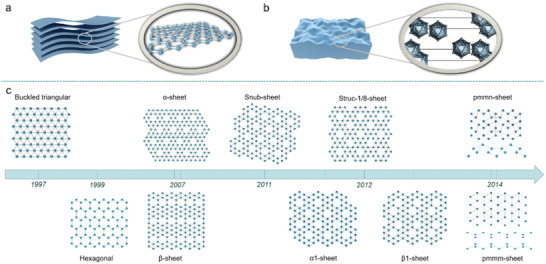
Different types of 2D materials’ bulk precursors. a) Structure of bulk graphite, of which the layer is connected by the van der Waals force. b) Structure of bulk boron, of which the structural units are combined through complicated bonding. c) Development history of borophene in theoretical models. Buckled triangular structure of borophene was first predicted in 1997; then a novel boron single layer structure, *α*‐sheet, consisting of triangular lattice and hexagonal honeycomb holes, was obtained through calculation. Boron atoms of *α*‐sheet are in the same plane, the energy of which are lower than the buckled triangular structure that was considered to be the most stable in previous studies. Based on this, during 2007–2014, a series of classical borophene structures such as *β*‐sheet, snub‐sheet, *α*1‐sheet, struc‐1/8‐sheet, *β*1‐sheet, *Pmmn*‐sheet, *Pmmm*‐sheet were occurred through the different arrangement of hexagonal and triangular lattices. The energy of these structures is very low and almost equal.

Due to the lack of electrons in the outermost layer, boron atoms can easily form chemical bonds; therefore, boron is chemically active and forms compounds with a variety of crystal structures. The main crystal structure of bulk boron (**Figure** **1**b) is based on a B_12_ icosahedron as the basic structural unit, with the various types of boron crystals being formed through different connections and bonding methods on the B_12_ icosahedron.^[^ ^40^ ^]^ Therefore, the boron block is not a layered structure like graphene (Figure 1a), making the preparation of borophene complex and difficult. In 2006, Kunstmann and Quandt^[^ ^41^ ^]^ first predicted the basic structure of 2D boron sheets, unveiling the mystery of borophene. In 2014, Wang et al.^[^ ^42^ ^]^ demonstrated for the first time that B_36_ is an extremely stable quasi‐planar borophene cluster with a hexagonal hollow structure, confirming that it is feasible for monoatomic layer borophene clusters to have hexagonal cavities. Wang et al. also presented the concept of borophene for the first time. In recent years, the theoretical research of borophene has achieved fruitful results, yet the synthetic methods and applications of boron remain few. The first synthesis of borophene was achieved in the laboratory in 2015.

Borophene has a variety of structures, classified according to the composition patterns—a) distorted hexagonal (DH) plane, b) buckled triangular (BT) plane, and c) mixed triangular‐hexagonal (MTH) plane, or to coordination number (CN)—*α* type (CN = 5, 6), *β* type (CN = 4, 5, 6), *χ* type (CN = 4, 5), *δ* type (CN = m, m is a single number), and *ψ* type (CN = 3, 4, 5).^[^ ^43^ ^]^ The DH plane (Figure 1c hexagonal structure) is a quasi‐planar structure composed of twisted hexagons, similar to graphene.^[^ ^44^ ^]^ The BT plane (Figure 1c buckled triangular structure) is a plane with B_7_ as the basic unit. B_7_ is a pyramid‐like structure formed through the positioning of a boron atom in the middle of a boron atom hexagon.^[^ ^36^, ^43^ ^]^ The central boron atom divides the hexagon into six small triangles, such that the triangle is also the basic unit of the BT plane. The MTH plane (Figure 1c *α*‐sheet) consists of a hollow hexagon motif (HHM) and a triangular motif (TM), with an adjustment of their ratio leading to a variety of different MTH planes.^[^ ^45^, ^46^, ^47^, ^48^, ^49^, ^50^ ^]^ Combining the two classifications, we can find that both the DH plane and the BT plane belong to the *δ* type plane, and the MTH plane includes all planes except the *δ* type.

Polymorphism is one of the most important characteristics of borophene, due to the complex bonding mechanism between boron atoms and the quite close formation energies of freestanding borophene. In 2006, Kunstmann et al.^[^ ^41^ ^]^ first predicted the basic structure of 2D boron according to the Aufbau principle, and proposed the buckled structure with B7 as the basic unit (Figure 3a), which is the main structure of borophene in the early theoretical studies. In 2007 Tang et al. predicted one of the most classic energy‐dominant structure *α*‐sheet.^[^ ^51^, ^52^ ^]^ Boron atoms of *α*‐sheet are in the same plane, of which the energy is 0.12 eV per atom, lower than the buckled triangular structure that was considered to be the most stable in previous studies. It can be considered as removing atoms from the flat triangular lattice; that is, based on the buckled triangular (BT) plane, the hollow hexagonal hole can be obtained by removing the central boron atom of the B7 basic unit, thereby creating the mixed structure of hexagonal holes and triangular lattices.^[^ ^43^ ^]^ The *α*‐sheet can be seen as the expansion of B_80_, with all boron atoms in the same low‐energy plane. The number and distribution of hollow hexagons (HHs) determine the different structures of borophene. For a better understanding, a hexagon hole density (*η*) has been developed, wherein

(1) $$\eta =\frac{\mathrm{No}.\;\mathrm{of}\;\mathrm{hexagon}\;\mathrm{holes}}{\mathrm{No}.\;\;\mathrm{of}\;\;\mathrm{atoms}\;\mathrm{in}\;\mathrm{the}\;\mathrm{original}\;\mathrm{triangular}\;\mathrm{sheet}}\;$$

The value of *η* and the pattern of hexagon holes determine the energy of 2D boron, and the self‐doping system can maintain the optimal stability by rationalizing *η*. According to the formula, *η* of BT plane is 0, DH plane is 1/3, *α*‐sheet is 1/9.^[^ ^53^ ^]^

However, since there are numerous arrangements of hexagonal holes in borophene, it is unrealistic to predict every possible structure by using density functional theory (DFT) method directly. Yakobson et al.^[^ ^47^ ^]^ treated borophene as the pseudo‐alloy B_1−*ν*[]*ν*_, composed of hexagonal holes and triangular lattices, so as to effectively assess the diversity and structural stability, where the vacant sites in the closed‐packed triangular lattices are named as "[ ]", and *ν* = *m*/*N* represents the hexagonal holes concentration and the quantities of hexagonal holes in a supercell of *N* lattice positions are represented by *m*.^[^ ^37^ ^]^ By theoretical calculation, they also found that borophene is relatively stable when the hexagonal holes concentrations is between 10% and 15%, and the cohesive energies of borophene with different structures are extremely low and close at the narrow range of hexagonal holes concentrations just mentioned.

Based on these theoretical studies, more stable structures, such as g1/8‐sheet, g2/15‐sheet, *α*1‐sheet, *β*1‐sheet, snub‐sheet, struc‐1/8‐sheet, *Pmmn*, and *Pmmm*, have been successively predicted.^[^ ^28^, ^43^, ^47^, ^53^, ^54^ ^]^ Figure 1c shows their specific structures according to the year of discovery. Borophene obtained experimentally is often composed of various structures due to the energies of the thermodynamically stable structures being similar.

## 3 Synthesis of Borophene

**Figure 2:**
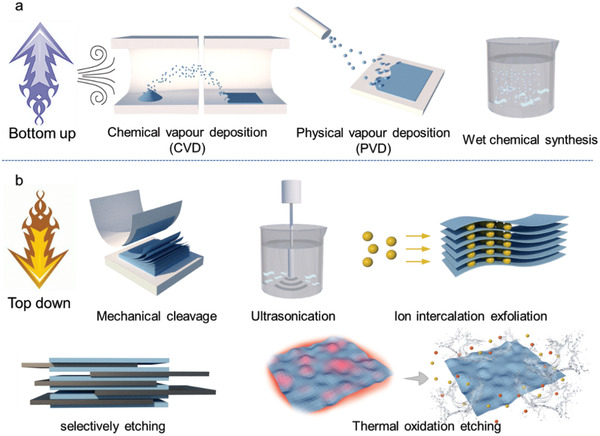
The main synthesis method of 2D nanosheets. a) Bottom‐up method: CVD, PVD, and wet chemical. b) Top‐down method: mechanical cleavage, ultrasonication, ion intercalation exfoliation, selectively etching, and thermal oxidation etching.

“Bottom‐up” and “top‐down” techniques are two main methods for the preparation of 2D nanomaterials. The “bottom‐up” approach includes physical vapor deposition (PVD),^[^ ^55^, ^56^, ^57^ ^]^ chemical vapor deposition (CVD),^[^ ^58^, ^59^, ^60^, ^61^, ^62^, ^63^, ^64^, ^65^ ^]^ and wet chemical synthesis methods,^[^ ^66^, ^67^, ^68^, ^69^ ^]^ amongst others (**Figure** **2**a).^[^ ^70^ ^]^ CVD involves the formation of a film on a substrate through related gas phase precursors reacting on it. The environment, substrates, and precursors are three crucial factors affecting the properties and growth of the products in this method. Metal oxides, graphene, and h‐BN have all been synthesized by CVD. During the process of PVD, the material source is vaporized into a gaseous state through physical method under vacuum conditions and deposited onto a substrate to form a functional film. 2D nanosheets of non‐layered materials are often prepared by chemical reactions in solution, termed wet chemical synthesis. Many materials, such as CuS, Co_3_O_4_, *α*‐Fe_2_O_3_, WO_3_, and Rh, have been prepared by this high‐yield and low‐cost method. In general, bottom‐up approaches can be used for the synthesis of almost all types of 2D nanomaterials. “Top‐down” approaches include mechanical cleavage,^[^ ^71^, ^72^ ^]^ ultrasonication,^[^ ^73^ ^]^ ion intercalation exfoliation,^[^ ^74^, ^75^, ^76^, ^77^ ^]^ and etching^[^ ^78^, ^79^, ^80^ ^]^ (Figure 2b). Mechanical cleavage relies on shear forces to overcome the van der Waals forces between connection layers and is widely used in the exfoliation of 2D materials from bulk materials.^[^ ^7^, ^72^ ^]^ Of note, the first successful synthesis of graphene was through this method.^[^ ^8^ ^]^ Ultrasonication induces liquid exfoliation depending on shear forces; its acoustic energy can delaminate the layered materials such as graphite and MoS_2_. In this method, the size, quality, and dispersion of 2D materials can be controlled by sonication solvent and time. The advantages of ultrasonication are low cost and large production scale, but the yield of single layer 2D material is low. During ion intercalation‐exfoliation, ions intercalate into layer spacing to reduce the van der Waals forces between layers, inducing the exfoliation. Layered material, including transition‐metal dichalcogenides (TMDs) and graphite, can be easily exfoliated by this method. Top‐down approaches mentioned above are mainly suitable for van der Waals solids, while etching methods such as acid etching and thermal oxidation etching are commonly used in non van der Waals solids. Selectively etching and exfoliation are utilized to prepare 2D materials with strong interlayer bond in their bulk materials, which cannot be obtained by simple mechanical exfoliation. 2D material Mxene can be obtained through selectively etching the A layers of MAX (Mxene's precursor) by HF. In the process of thermal oxidation etching, the material oxidizes rapidly at high temperature and forms oxides on the surface, which are unstable in air or water. Therefore, substances on the surface of material can be oxidized away and nanosheets can be obtained through multiple oxidation and exfoliation. Borophen and g‐C3N4 were successfully prepared by this method.

### 3.1 Bottom‐Up Synthesis

#### 3.1.1 Synthesis on Ag(111)

**Figure 3:**
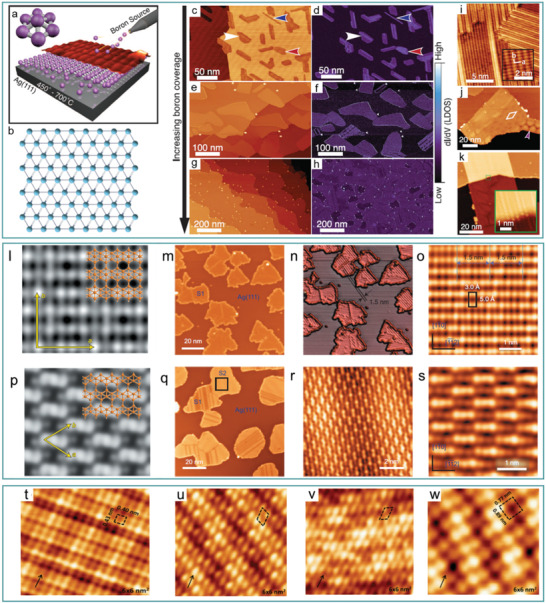
Synthesis and characterization of borophene. a) Schematics of synthesizing borophene on silver. a) Reproduced with permission.^[^ ^36^ ^]^ Copyright 2015, The American Association for the Advancement of Science. b) Structure of borophene predicted by computer. c–h) Closed‐loop d*I*/d*V* images of borophene on the right and large‐scale STM topography of borophene on the left, displaying low (c,d), medium (e,f), and high (g,h) coverage. The blue, red, and white marks denote striped‐phase nanoribbons, striped‐phase islands, and homogeneous‐phase, respectively. i) STM images about atomic‐level structure of striped‐phase. The rectangular lattice shown in the illustration has overlaid lattice vectors. j) The purple arrow and white rhombus denote honeycomb and rhombohedral Moiré patterns in striped phase, respectively. k) Carpet‐mode growth demonstrated by striped‐phase island. The illustration indicates that atoms across Ag (111) step have continuity. c–k) Reproduced with permission.^[^ ^36^ ^]^ Copyright 2015, The American Association for the Advancement of Science. l) Simulated STM image of *β*12 sheet. m) STM image about borophene on Ag (111) substrate. n) 3D image of (m), and the 1.5 nm stripes can be clearly seen. o) High‐resolution STM image about S1 phases. p) Simulated STM image about *χ*_3_ sheet. q) STM image of borophene on Ag (111) (650 K); most of the borophene islands are transformed from “S1” phase to “S2” phase. r) STM image of the area of (f) (highlight by the rectangle). s) High‐resolution STM image about (r) (“S2”). l–s) Reproduced with permission.^[^ ^13^ ^]^ Copyright 2016, Springer Nature. t–w) High‐resolution STM images for P1–P4 of boron nanoribbons on Ag (110), respectively. t–w) Reproduced with permission.^[^ ^81^ ^]^ Copyright 2017, American Physical Society.

In 2015, Guisinger et al.^[^ ^36^ ^]^ successfully synthesized ultrathin monoatomic layer borophene on clean silver at temperatures between 723 and 973 K, under ultrahigh vacuum conditions by using boron vapor (99.9999% purity, boron flux is controlled at 0.01 to 0.1 monolayer per minute [ML min^−1^]) as the boron source (**Figure** **3**a,b). The high‐purity source of boron atoms avoided the use of toxic precursors (e.g., diborane) and the clean silver substrate provided a good inert surface for the growth of borophene. This method led to the formation of two phases of borophene; homogeneous phase and more corrugated “striped” phase were formed on silver at 820 K (Figure 3c,d). Both the temperature and deposition rate were shown to affect the relative concentrations of the two phases, wherein low deposition rates favor the generation of striped phase whereas higher deposition rates favor the generation of homogeneous islands (Figure 3c–h). Furthermore, the formation of the striped phase was closely related to temperature, with a minimal presence of this phase at 720 K and an almost exclusively striped phase at 970 K (atomic‐scale structure and growth mode of striped phase shown in Figure 3i–k). The authors also characterized the ultrathin monoatomic layer borophene material by Auger electron spectroscopy (AES), scanning tunneling microscopy (STM), and aberration‐corrected scanning transmission electron microscopy (AC‐STEM) in this study, and showed that borophene had metallic properties along the direction of the stripe and a bandgap along the out‐of‐plane fold orientation, as proposed based on the calculated band structure. Thus, borophene is a highly anisotropic 2D material able to conduct electricity along the stripe direction. In addition, due to the strong interactions between boron atoms, the Young's modulus in the out‐of‐plane wrinkle direction is higher than that of graphene, resulting in the anisotropic mechanical properties of the wrinkled borophene structure. Therefore, it is likely that 2D borophene with metallic properties and a high mechanical strength will play an important role in related applications.

In the same period, Wu et al.^[^ ^13^ ^]^ grew monoatomic layer borophene on a Ag(111) substrate using boron with a purity of 99.9999% as the boron source under ultrahigh vacuum conditions. Two borophene phases, termed S1 (Figure 3l–o) and S2 (Figure 3p–s), were observed corresponding to the theoretical predictions *β*_12_ and *χ*_3_. Both of these phases had triangular lattices, albeit with differing arrangements of the periodic holes. Additionally, temperature influenced the formation of borophene—the S1 and S2 phases were preferably formed at 570 and 650 K, respectively, while between 650 and 800 K, the S1 and S2 phases coexisted. Furthermore, when boron coverage was close to a monolayer, 3D boron clusters began to form, indicating that the boron–silver interaction played an important role in the growth of monolayer borophene.

In 2017, after successfully obtaining borophene on Ag (111), Chen and Wu et al.^[^ ^81^ ^]^ synthesized high‐quality and good‐uniformity borophene nanoribbons (BNRs) on Ag (110) with width concentrated around 10 nm at a temperature of 570 K. There are four phases on the BNR, which are P1, P2, P3, and P4. The unit cells of P1 and P4 are both rectangular; the atomic models for them can be explained by *χ*_3_ and *β*_8_ sheet, respectively, with the lattice constants of *a* = 0.40 nm, *b* = 0.43 nm for P1 and *a* = 0.89 nm, *b* = 0.77 nm for P4 (Figure 3t,u). The unit cell of P2 and P3 are rhomboid; both of them can be explained by *β* structure model, with the lattice constants of *a* = 0.44 nm, *b* = 0.82 nm for P2 and *a* = 0.45 nm, *b* = 0.76 nm for P3 (Figure 3v,w). However, the structures mentioned above are all based on the boron chains that may exist deviations, and in order to make it more accurate to understand the structures of BNPs, Chen and Wu et al. designed a novel notation, BC (*n*, *m*), where the quantities of atoms in a single boron chain are defined by m (the narrowest regions) and n (the widest regions). According to this notation, *β*(P2, P3), *χ*_3_(P1) and *β*_8_(P4) can be described as BC(3,4), BC(2,2), and BC(4,4), respectively.

#### 3.1.2 Synthesis on Cu(111)

**Figure 4:**
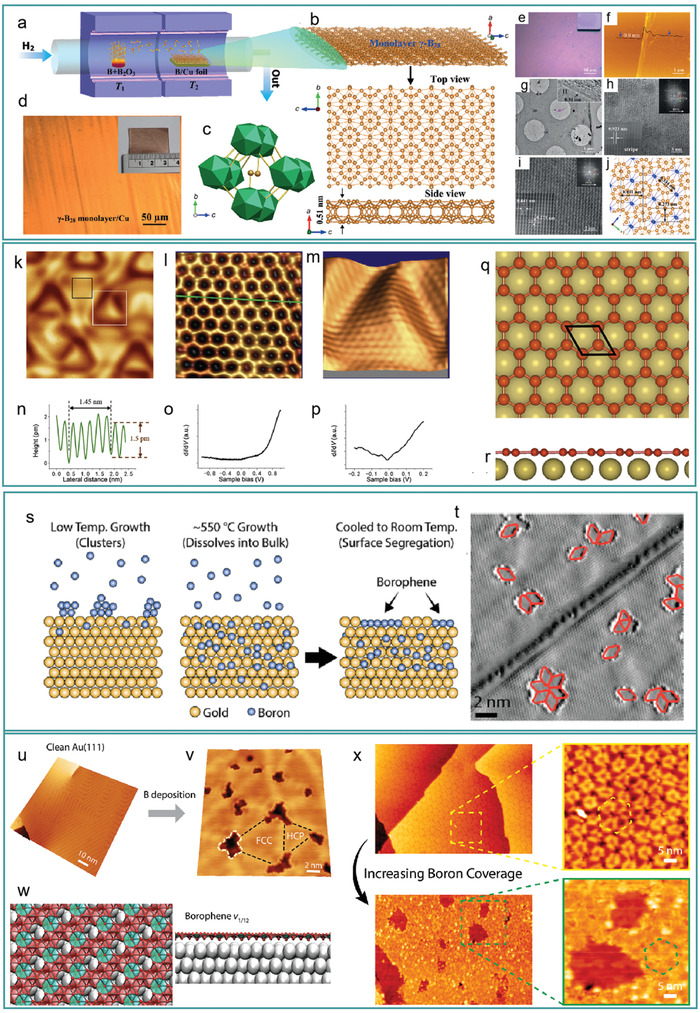
a) Schematic of CVD synthesis of 2D *γ*‐B_28_ films on Cu foil by using two‐zone furnace. b) Structural of borophene on Cu foil. c) Structure of the basic unit cell of 2D *γ*‐B_28_. d) Optical image and photograph of borophene on Cu foil. e) Optical image and photograph of *γ*‐B_28_ on SiO2/Si substrate (thickness: 285 nm). f) AFM topographical image of borophene on SiO_2_/Si substrate. Height along the black line is 0.80 nm, indicating monolayer film was made. g) Low‐resolution TEM image of the film. 1L: one layer. h) HRTEM image of thin area of the film. Inset: Image on top right are FFT and image on bottom left corners are reconstructed FFT images. i) HRTEM image of the thick area of the film, indicated by the pink arrow in (g). Inset: The imagesatthe top right are the FFTs and the imagesat the bottom left corners are reconstructed FFT images. j) Structure of *γ*‐B_28_ (orange indicates anionic and blue indicates cationic). a–j) Reproduced with permission.^[^ ^82^ ^]^ Copyright 2015, Wiley‐VCH. k) STM image about borophene with triangular corrugation on Al (111). l) A high‐resolution STM image about an area of (k) (highlightedby the black square), in which the honeycomb lattice can be clearly seen. m) 3D STM image about an area of (k) (highlightedby the white square). n) The profile of the green line in (l). o,p) d*I*/d*V* curves of borophene under different bias voltages. q,r) The hexagonal honeycomb structure of borophene on Al (111): q) top views and r) side views. k–r) Reproduced with permission.^[^ ^84^ ^]^ Copyright 2018, Elsevier. s) Schematic diagram of borophene synthesis on Au (111). t) STM image of the borophene nanostructures in initial stages of growth. u) STM image of an Au (111) surface with herringbone reconstruction. v) Trigonal network appeared on Au(111) at 823 K and borophene islands (shown by the white line) formed at the nodes of the trigonal network. w) Borophene *ν*_1/12_ on Au (111) predicted by computer (top view on the left and side view on the right). x) The trigonal network was broken and turned into larger borophene islands when the boron dose increased. s–x) Reproduced with permission.^[^ ^85^ ^]^ Copyright 2019, American Chemical Society.

Wang et al.^[^ ^82^ ^]^ used a homemade two‐part CVD boiler to synthesize borophene (**Figure** **4**a). Prior to the synthesis of borophene, copper foil (25 µm thick) in T2 was heated to 1237 K, so as to enlarge the grain boundaries and smoothen the copper foil surface. Subsequently, the T1 zone was set to 1337 K, and B (99.99%) and B_2_O_3_ (99.98%) were placed at a 1:1 ratio in T1 to generate B_2_O_2_ vapor. Finally, hydrogen transported the B_2_O_2_ vapor into the T2 zone, and borophene was formed on the copper foil.

The structure of borophene synthesized by the above method was a 3D network composed of B_2_ dumbbells and icosahedral B_12_ (Figure 4b,c). Optical images of borophene elucidated that the film was large and continuous (Figure 4d,e). Atomic force microscopy (AFM) (Figure 4f) and transmission electron microscopy (TEM) images (Figure 4g) showed the borophene to have a folded edge thickness of 0.80 nm, indicating that the film was composed of a monolayer. High‐resolution transmission electron microscopy (HRTEM) images exhibited that borophene contains a 1D longitudinal stripe composed of two selectable lattice planes, with a pitch of 0.923 nm, demonstrating that the structure of borophene has *Pnnm* characteristics. The lattice structure of borophene was revealed by fast Fourier transform (FFT) and HRTEM images (Figure 4h,i). With a combination of the above characterization results with single‐crystal/powder X‐ray diffraction and first‐principles calculations, it was shown that monolayer _ϒ_‐B_28_ (Figure 4j) was successfully synthesized on copper by CDV.

Gozar et al.^[^ ^83^ ^]^ have synthesized borophene on Ag (111) and Cu (111), respectively, in 2019, and studied the synthetic characteristics for these two conditions and the crystal structure of borophene systematically, combining ab initio DFT, STM, diffraction, and low‐energy electron microscopy. They found that the domains of borophene on Ag (111) maintain nanoscale under all growth conditions such as various substrate temperatures. However, the nanoscale single‐crystal domains came out so far are too small to fabricate devices, while the larger the size of single‐crystal domain is, the more beneficial borophene application in devices will be. Furthermore, it was reported that the metal substrate materials can influence film adhesion and rippling, growth dynamics, and other properties of borophene. What is said above illustrates the chosen metal substrate material is fairly crucial for device fabrication. Surprisingly, Gozar et al. proved that Cu (111), less inert than Ag, is more conductive to larger‐size domains growing in theory, and they have obtained large single‐crystal domains of borophene, up to 100 µm^2^ in size, on the Cu (111) surface at *T* = 770 K and at a rate of 0.05 ML min^−1^.

#### 3.1.3 Synthesis on Al(111)

Theoretical calculations showed that, due to the absence of electrons, the main structure of borophene is a triangular dense packed lattice accompanied by HHs, and that the formation of a honeycomb hexagonal borophene structure similar to that of graphene is extremely difficult. However, such a unique 2D hexagonal honeycomb structure would endow borophene with a Dirac fermion band structure, exhibiting the quantum effects found in grapheme such as emergence of the quantum Hall effect, massless fermions, and extremely high electron mobility. These properties would promote a series of applications for borophene, including as biosensors and nanoelectronic materials. The well‐known superconducting material MgB_2_ is formed by the alternate arrangement of boron atomic layers with a hexagonal honeycomb structure and magnesium atomic layers with a triangular close‐packed structure. Additionally, its superconductivity is derived from electron–phonon coupling, in which the metallic boron atomic layer plays a key role. Therefore, the successful synthesis of borophene with a honeycomb structure would be of particular interest.

Wu et al.^[^ ^84^ ^]^ achieved a major breakthrough in the synthesis of borophene with a long‐awaited planar hexagonal honeycomb structure. Monoatomic layer borophene was grown on an Al (111) substrate using boron vapor with a purity of 99.9999% under ultrahigh vacuum conditions at a deposition rate of 0.1 ML min^−1^ (temperature: 500 K).

Atoms of borophene appear equivalent, indicating that the honeycomb lattice was locally flat (Figure 4k,l,n,q,r); however, the honeycomb lattices are overlapped, with atomic corrugation less than 1.5 pm on the large‐periodic (Figure 4m).

Its d*I*/d*V* curves indicated that the hexagonal honeycomb structure borophene had metallic properties (Figure 4o). Furthermore, the V‐shaped d*I*/d*V* curve indicated that hexagonal honeycomb structure borophene had a dip Fermi energy similar to the characteristic Dirac bands of graphene (Figure 4p).

#### 3.1.4 Synthesis on Au(111)

Recently, Guisinger et al.^[^ ^85^ ^]^ successfully synthesized borophene on an Au(111) substrate and explored the influence of Au (111) substrates with different temperature on the thermal deposition of boron atoms. SEM images of clean Au (111) and borophene demonstrated the successful synthesis of borophene on Au (Figure 4u,v). Unlike the synthesis of borophene on Ag(111), Cu(111), and Al(111) substrates, on the Au(111) substrate, boron atoms first dissolved into gold atoms at high temperatures (equal or greater than 823 K) and segregates to the surface to form borophene after cooling.(Figure 4s). In the process of borophene synthesis on a Au(111) substrate, nanoscale borophene islands were observed on the surface of Au(111) (Figure 4v), composed of 1–8 rhombic structures of 1 nm^2^ in surface area with a borophene *ν*_1/12_ atomic structure and metallic conductivity (Figure 4t,w). With the increase in boron concentration, the trigonal network was broken down, forming larger borophene islands on the substrate surface (Figure 4x). Therefore, the larger the boron atom concentration, the larger the size of borophene, based on the current studies. However, the further applications of borophene are limited due to the difficulty of transferring borophene from metal substrates. Due to the weak bonds and limited charge transfer between Au(111) and borophene formed on the surface, borophene was easily peeled off, which will allow further application.

However, only a few reports are available regarding the synthesis of borophene in the laboratory. All of the above examples of successful borophene synthesis reported thus far involve bottom‐up methods except for two reports from 2018, in which top‐down methods were used, as described below.

### 3.2 Top‐Down Synthesis

**Figure 5:**
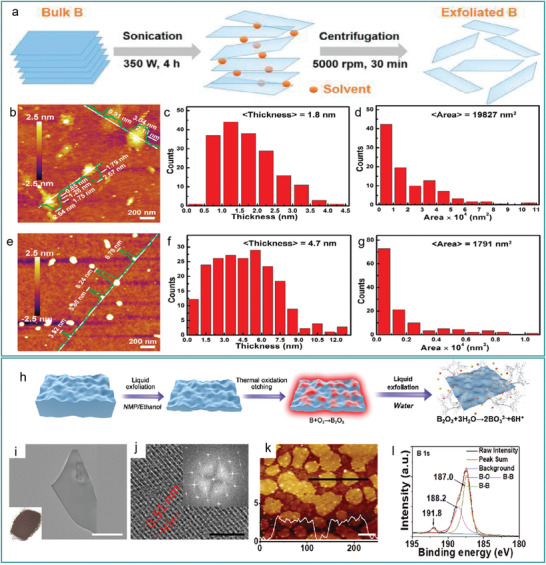
a) Schematic of the sonication‐assisted liquid‐phase exfoliation synthesis of high‐quality borophenes. b–g) AFM characterization of borophene synthesized in DMF (b–d) and IPA (e–g) by sonication. b,e) AFM topographic images and height profiles. c,f,d,g) Thickness and size of prepared borophene. a–g) Reproduced with permission.^[^ ^86^ ^]^ Copyright 2018, American Chemical Society. h) Schematic of thermal oxidation etching and liquid exfoliation synthesis of borophene. i–l) TEM, HRTEM, AFM images, and XPS spectra of borophene synthesized by liquid exfoliation in water. The white scale bar = 100 nm and the black scale bar = 5 nm. h–l) Reproduced with permission.^[^ ^25^ ^]^ Copyright 2018, Wiley‐VCH.

Teo et al.^[^ ^86^ ^]^ proved that high‐quality multilayer borophene with controlled size and thickness can be synthesized by ultrasonically assisted liquid‐phase exfoliation. During the synthesis process, boron powder was dispersed in a dimethylformamide (DMF)/isopropyl alcohol (IPA) solvent (1 mg mL^−1^) and sonicated at 350 W for 4 h. Borophene of different thicknesses was collected by adjusting the centrifuge speed (**Figure** **5**a). Subsequently, the obtained borophene was characterized by AFM (Figure 5b,e). Borophene stripped in DMF had an average thickness of 1.8 nm and an area of 19 827 nm^2^ (Figure 5c,d), while borophene peeled off in IPA had a thickness of 4.7 nm and an area of 1791 nm^2^ (Figure 5f,g); thus, the size and thickness of borophene can be controlled by adjusting the solvent used in ultrasonic exfoliation.

Inspired by the special chemical properties of boron, in which borophene showed inertness to oxidation, whereas its outer edge and 3D bulk boron were both easy to oxidize, Ji et al.^[^ ^25^ ^]^ developed another top‐down synthesis method using high‐temperature etching and liquid‐phase stripping. The boron powder was dispersed in an N‐methyl pyrrolidone (NMP) and alcohol 1:1 solvent and ultrasonicated for 5 h at 500 W. Next, boron flakes were separated and placed in an oxygen‐containing ceramic vessel at 923 K to oxidize the surface of the boron flakes to B_2_O_3_. Subsequently, a liquid‐phase exfoliation method was used to make the surface of B_2_O_3_ dissolve into BO_3_^3−^ (Figure 5h). Finally, borophene with a planar size of ≈110 nm and thickness <3 nm was obtained (Figure 5i,k). Borophene contained interference fringes, and its stripe pitch was 0.51 nm, matching the characteristics of a *β*‐rhombohedral boron structure (Figure 5j). XPS spectra of borophene (Figure 5l) further confirmed the successful synthesis of borophene following the exfoliation of oxidized layers in water.

This work is the first report discussing the synthesis of boron bulks into borophene by a top‐down method, greatly improving the specific surface area of borophene, and improving its application in drug delivery.

### 3.3 Key Factors in the Synthesis of Borophene

Based on the results of theoretical research and experimental synthesis, the substrate, temperature, and solvent have been shown to be the key factors affecting the formation of borophene. Next, we will analyze these key factors in detail.

#### 3.3.1 Effect of Metal Substrate on Borophene Synthesis

Classical nucleation theory shows that the nucleation energy barrier regulates the material formation, whereas the 2D boron nucleation barrier is higher than its 3D bulk structure, preventing the natural nucleation of borophene.^[^ ^87^, ^88^, ^89^, ^90^, ^91^ ^]^ The thermodynamic defects and polymorphism of borophene increases the difficulty of its synthesis. Therefore, reducing the 2D boron nucleation barrier and inducing 2D nucleation is an energetically favorable route conducive to borophene synthesis.^[^ ^92^ ^]^ In 2013, Yakobson et al.^[^ ^87^ ^]^ indicated that the interaction between metal substrates and boron helps the nucleation of boronene because the 3D nucleation barrier of boron on some metal substrates is higher than 2D. What kind of substrate is suitable for borophene synthesis? Two criteria have been suggested for the selection of appropriate metal substrates—it should have a slight solubility to boron atoms and the binding force between the substrate and the boron atoms should be moderate.^[^ ^92^ ^]^ If the force is too strong, it is difficult to peel borophene off from the substrate and borophene may even easily form a boride. For example, when borophene is synthesized on Fe, Co, or Ni substrates,^[^ ^93^, ^94^ ^]^ boride can be easily formed. Additionally, on Au substrates, since the interaction between Au and boron atoms is too weak, non‐planar boron may easily form.^[^ ^95^ ^]^

According to the interaction between borophene and the metal substrate, the commonly used substrates can be divided into two major categories: one has strong binding energy and large charge transfer with borophene, including Mg(0001), Al(111), and Ti(0001).^[^ ^96^, ^97^ ^]^ The profile of *σ* (in‐plane) bands of hexagonal borophene synthesized on these substrates is similar to its freestanding phase, except for a Fermi level shift. The other has a relatively lower binding energy with borophene, including Au(111) and Ag (111), and the in‐plane bands of hexagonal borophene split into many sub‐bands.^[^ ^98^ ^]^ Xu et al.^[^ ^99^ ^]^ theoretically assessed the nucleation mechanism and growth process of borophene on the surface of Ag(111), and confirmed that the optimal stable structure was one where the HHs are arranged in parallel and the concentration of HHs was 1/6 by experiments. Zhao et al.^[^ ^100^ ^]^ found that 2D dense boron clusters were easily formed on Cu(111) surface. Furthermore, the larger the cluster size, the smaller the formation energy.

Thus, the metal substrates make a great difference on the structure of the formed borophene, and it is particularly important to select a suitable substrate according to the target product.

#### 3.3.2 Effect of Temperature and Deposition Rate on Borophene Synthesis

In theory, the 3D structure of boron is more stable than borophene, and is therefore more easily formed. Therefore, the induction of boron atoms to prefer 2D nucleation is one of the key points in the synthesis of borophene. The external growth environment has a great impact on the nucleation barrier of materials, affecting the free energy of boron growth—only when the free energy is higher than the nucleation barrier can boron atoms grow to a specific structure. At low temperature, the desired 2D structure cannot be obtained because the free energy of boron growth is much lower than the nucleation barrier of borophene. Similarly, when the temperature is too high, boron atoms tend to grow into a 3D structure because the free energy of boron growth is much higher even exceeding the 3D boron nucleation barrier. Therefore, in order to synthesize borophene, the temperature needs to be strictly controlled so that the free energy of boron growth is maintained between the energy region of the 2D and 3D nucleation barrier. Furthermore, Wu et al.^[^ ^13^ ^]^ respectively proved that different phases of borophene could be synthesized at different temperatures. Additionally, the real‐time control and detection of the atomic deposition rate are key in the control of crystallization quality and borophene coverage. Guisinger et al.^[^ ^36^ ^]^ found that a lower deposition rate was beneficial to the formation of a fringe phase (*β*_12_). Gozar et al.^[^ ^83^ ^]^ found that the deposition rate had a major influence on the crystallization of 2D materials as observed by the synthesis of borophene in Ag(111). The crystallinity of the material would decrease when continuous deposition exceeds 1 ML coverage. Therefore, it is necessary to strictly control the deposition rate and the amount of raw materials according to the actual needs.

#### 3.3.3 Effect of Solvents on Borophene Synthesis

In the top‐down synthesis method, temperature does not have a great influnce on the formation of borophene, whereas the size and thickness of borophene can be directly determined by changes in the selected solvent.^[^ ^101^ ^]^ The polarity of the solvent determines the exfoliation mechanism of borophene, which in turn affects the size, thickness, and layers. Vinu et al.^[^ ^102^ ^]^ successfully synthesized borophene through sonochemical exfoliation and explored the effect of different solvents (acetone, ethylene glycol, water, isopropyl alcohol, and DMF) on the synthesis of borophene. According to Vinu's study, EG and acetone tend to intercalation‐mediated exfoliation and are conducive to yield borophene with monolayers, wherein acetone performs better in producing borophene monolayers. On the contrary, other solvents can rarely yield monolayers because of their different exfoliation mechanism—DMF (yield multilayered borophene) and water (yield few monolayers) tend to mediate the surface exfoliation of borophene, while isopropyl alcohol (yield few monolayers) tends to fragment exfoliation.

Thus, the structure and physicochemical properties of borophene are affected by the synthesis methods and conditions. In order to facilitate the selection of suitable methods according to the practical application needs, we provide a comparative analysis and summary of the various synthesis methods (**Table** **1**).

**Table 1:** Comparison of different preparation methods

Method^a)^		*T* [K]	Substrate	Structure	Materials	Deposition rate [ML min^−1^]	Conditions	Costs	Area	Thickness	Ref.
Bottom‐up	MBE	720 970	Ag (111)	Homogeneous phase (720 k), stripe phase (970 k)	Pure boron 99.9999%	0.01–0.1	Ultrahigh vacuum	High	Small	Single layer	^[^ ^36^ ^]^
	MBE	570 650	Ag (111)	*β*_12_ (570 k), *χ*_3_ (650 k)	Pure boron 99.9999%	—	Ultrahigh vacuum	High	Small	Single layer	^[^ ^13^ ^]^
	MBE	500	Al (111)	h‐BS (honeycomb borophene)	Pure boron 99.9999%	0.1	Ultrahigh vacuum	High	Small	Single layer	^[^ ^84^ ^]^
	MBE	823	Au (111)	Borophene *ν*_1/12_	Pure boron 99.9999%	0.02	Ultrahigh vacuum	High	Small	Single layer	^[^ ^85^ ^]^
	CVD	1373 1273	Cu (111)	*γ*‐B_28_	Boron (99.99%) and B_2_O_3_ (99.99%)	—	Low pressure	High	Large	0.80 nm	^[^ ^82^ ^]^
Top‐down	Thermal oxidation etching and liquid exfoliation	923	—	*β*‐rhombohedral B structure	Boron powder	—	Ordinary pressure	Low	Small	<5 nm	^[^ ^25^ ^]^
	Sonication‐assisted liquid phase	—	—	*β*‐rhombohedral B structure	Boron powder (95%)	—	Ordinary pressure	Low	Small	1.8–4.7 nm	^[^ ^86^ ^]^

a) MBE, molecular beam epitaxy; CVD, chemical vapor deposition.

## 4 Properties of Borophene

### 4.1 Chemical Property

2D boron has been shown to be inert to oxidation, whereas its outer edge and 3D bulk boron are less so.^[^ ^13^ ^]^ Boron has three valence electrons ([He]2S^2^2P^1^); however, an electron promoted from the 2S to the 2P orbital can enable boron to have four available valence orbitals. Therefore, the number of valence electrons is less than the number of valence orbitals and the electronic orbitals cannot be fully filled with boron atom electrons when forming chemical bonds, leading to the formation of electron‐deficient boron atoms. The conjugations of bulk 3D boron atoms and those of 2D boron atoms around the periphery are both based on classic two‐center two‐electron bonds, but the inner atoms of 2D boron are delocalized multicenter two‐electron bonds, resulting in their difference in oxidation stability.^[^ ^37^ ^]^ For example, in a boron sheet, the HHs serve as “acceptors” and the boron atoms at the center of hexagons act as “donors,” thus making up for the lack of electrons in boron and making boron sheets inert to oxidation.^[^ ^51^ ^]^

Wu et al.^[^ ^13^ ^]^ used X‐ray photoelectron spectroscopy (XPS) to detect the chemical composition of monolayer borophene, showing that the two low‐binding energy peaks (188.2 and 187.1 eV) are from two different B–B bonds in the 2D boron sheets, while the binding energy of the bulk boron 1s peak in 3D bulk boron is ≈189–190 eV generally. However, the higher energy peak (191.5 eV) is supposed to arise from the oxidized boron atom, based on oxidized boron peaks reported previously. Through the calculation and comparison of peak area, it was observed that the ratio of oxidized boron atom to unoxidized boron atom is only ≈0.23, indicating that most of boron atoms on the surface of borophene have certain stability against oxidation. Furthermore, the oxidized atoms are mainly located at the edge of borophene film, which can be verified by in situ STM experiments. Oxygen was pumped directly into the vacuum chamber and the surface of boron was scanned in situ using STM. At high oxygen concentrations, bright spots appeared at the edges of the boron island, indicating that the boron atoms began to be oxidized, while the terrace of boron sheets showed little change. As oxygen continued to flow in, the boron sheets remained stable and the striated phase was still visible, indicating that the oxidation of boron mainly occurs at the edges, while the boron sheet itself is stable against oxidation. Thus, 2D borophene is more inert to oxidation compared with 3D bulk boron.

In addition, Guisinger et al.^[^ ^36^ ^]^ also found that the oxidation state was detected in borophene when it was exposed to ambient conditions, yet oxidation mainly occurred on the edges of borophene because of its active edge states. Despite the high‐dose exposure of oxygen, the regions with the perfect lattice remain intact almost.^[^ ^103^ ^]^ Nevertheless, Guisinger et al. also demonstrated that borophene is not as chemically stable as other 2D materials and can be susceptible to contamination when exposed to air for long time. This chemical stability, while not comparable to that of graphene, helps to solve the problem of the instability against oxidation in 2D materials. Furthermore, the stability of borophene against oxygen can be increased by, for example, sealing borophene from oxygen by covering other materials, and they also proved that a silicon/silica oxide capping layer can greatly impede the oxidation of borophene, leading to our further study on the oxidation resistance of 2D boron.^[^ ^37^ ^]^

On the other hand, with chemical activity of borophene's edges, it can catalyze hydrogen evolution reactions;^[^ ^95^ ^]^ this interesting property not only providing a new possibility for the development of alternative energy, but also opening up new pathways for the application of boron.

### 4.2 Mechanical Properties

Borophene, constructed from multicenter covalent bonds instead of the classic two‐center covalent bonds present in other ordinary 2D materials, promises exceptional mechanical properties.^[^ ^104^, ^105^, ^106^, ^107^ ^]^ With a bond energy of ≈5.90 eV,^[^ ^108^ ^]^ in‐plane *σ* covalent bonds are among the strongest in nature, resulting in the ultrahigh in‐plane strength and excellent structural stability of graphene. In the case of borophene, in addition to the classical covalent bonds giving crucial strength to the sheet, metallic‐like multicenter bonds endow the structure with potential fluidity generally.^[^ ^95^ ^]^ Additionally, as a 2D material, borophene is speculated to exhibit excellent flexibility due to its atomic thickness. Moreover, according to predictions, borophene will undergo a structural phase transition at a large strain, leading to a higher mechanical toughness. Additionally, Wang et al.^[^ ^109^ ^]^ proved that, since the B‐B bonds weaken when increasing the tensile strain, the Poisson's ratio along the out‐of‐plane direction is negative.^[^ ^110^ ^]^ The specific parameters are summarized in **Table** **2**.

**Table 2:** In‐plane stiffness, in‐plane Poisson's ratio, fracture strength, ideal tensile strength, and fracture strain of borophene

In‐plane stiffness [N m^−1^]	In‐plane Poisson's ratio	Fracture strength *σ* [GPa]	Ideal tensile strength [N m^−1^]	Fracture strain *ε*	Method	Ref.
170(Z), 398(A)	−0.02(Z), −0.04(A)				Ab initio	^[^ ^36^ ^]^
163(Z), 399(A)			12.2(Z), 20.9(A)	0.143(Z), 0.087(A)	Ab initio	^[^ ^107^ ^]^
163(Z), 382(A)	−0.01	33.6(Z), 54.8(A)		0.145(Z), 0.105(A)	Ab initio	^[^ ^105^ ^]^
166(Z), 389(A)		31.2(Z), 48.7(A)	12.98(Z), 20.26(A)	0.15(Z), 0.08(A)	Ab initio	^[^ ^109^ ^]^
163(Z), 394(A)	−0.01(Z), −0.03(A)	33.6(Z), 57.7(A)		0.15(Z), 0.10(A)	MD	^[^ ^106^ ^]^

It has been shown that the in‐plane stiffness of *ν*_1/6_ borophene is lower than many 2D materials, such as graphene.^[^ ^107^ ^]^ It was calculated that the Foppl–von Karman number per unit area of the *ν*_1/6_ sheet, which is defined as C/D, that is, the ratio between the in‐plane modulus and bending stiffness, is the highest among the materials mentioned in that research. Thus, it is one of the most flexible materials, with high levels of flexibility against off‐plane deformation. Borophene can be classified according to its atomic geometry, including triangular sheets (*ν*_0_), *α* sheets (*ν*_1/9_), *ν*_1/5_, *ν*_1/6_, *ν*_1/7_, *ν*_1/8_, *ν*_1/10_, and *ν*_1/12_ sheets.^[^ ^111^ ^]^ The relaxed triangular sheets present a washboard‐like buckling structure due to an excess of electrons, while the *ν*_1/5_, *ν*_1/6_, and other sheets are purely planar due to the high HH concentrations resulting in a lack of electrons in vacuum. The planar structure solves the instability caused by the buckling structure of triangular boron sheet. As mentioned above, the introduction of HHs into borophene can effectively stabilize the structure due to the applied strain being highly concentrated near HHs, which controls the elasticity of the whole sheets. On the other hand, the increasing HH concentration can expand the sheet area, thereby reducing tension, and the structural phase transition experienced by borophene can make the material tougher and resist large loads even in extreme tension; therefore, HHs are critical for the ductile breaking process of borophene. Nevertheless, HHs considerably affect the mechanical properties of materials. Increasing HHs will reduce the number of B–B bonds and thus the hardness of the material. The ultimate tensile strength of the boron lattice will also be greatly affected. In other words, by simply adding HHs, despite the increase in *η*, the changes of the stifness of borophene are limited. Due to the atomic displacement method,^[^ ^107^, ^109^ ^]^ *ν*_1/6_ has been predicted to have an ideal strength of 16.4 N m^−1^—despite not being as good as that for graphene and hexagonal boron nitride (h‐BN) sheets, it is above other 2D materials, such as MoS_2_^[^ ^112^, ^113^ ^]^ and phosphorene.^[^ ^114^ ^]^ Furthermore, bending stiffness and the Poisson's ratio are also highly dependent on the density of HH; therefore, these mechanical parameters can be flexibly changed according to the requirements of different applications.^[^ ^107^ ^]^ The instability mechanism of borophene was also assessed,^[^ ^109^, ^115^ ^]^indicating that the failure mechanism is elastic instability under a uniaxial strain along the zigzag direction, while for the uniaxial strain along the armchair direction and biaxial strain, the failure mechanism is attributed to the phonon instability. As a 2D material with unprecedented flexibility, high ideal strength and excellent elasticity borophene has great potential in the preparation of composite materials and flexible devices. These data indicate that the properties of borophene can be tailored by adjusting the concentration of HHs and mastering the instability mechanism of borophene.

### 4.3 Electrical Properties

With the electron‐deficient nature of B element, the most stable structure is a mixture of hexagonal and triangle structures^[^ ^52^ ^]^ with two and three center bonding, respectively.^[^ ^50^, ^116^, ^117^, ^118^, ^119^ ^]^ Although different from the stable structure of graphene, borophene also has relatively unique electrical properties such as metallicity, Dirac–Fermi effects, superconductivity, and semiconductivity.

#### 4.3.1 Metallicity

Yakobson et al.^[^ ^120^ ^]^ theoretically predicted that all polymorphs of borophene are metallic, different from the semiconductor or insulating properties of 3D bulk allotropes, of which only a small part can turn into metal under ultrahigh pressure.^[^ ^121^ ^]^ Subsequently, it was experimentally confirmed that the metallicity is determined by the p_z_ orbit.^[^ ^39^ ^]^ Wu et al.^[^ ^13^ ^]^ utilized scanning tunneling spectroscopy to measure the electronic states of the *β*_12_ and *χ*_3_ phases successfully synthesized, and found that there are local densities of states around the Fermi level, further demonstrating the energy band structure of metallicity. Matsuda et al.^[^ ^122^ ^]^ observed the metallic boron‐derived bands of *β*_12_ by using angle‐resolved photoelectron spectroscopy, and inferred an interesting phenomenon—the coexistence of electronic pockets and hole pockets at the Fermi level—according to the first‐principles calculations, which also indicates its metallicity. Furthermore, Fazzio et al.^[^ ^123^ ^]^ confirmed that the conductivity of borophene is anisotropic and the current transport is directionally dependent in two perpendicular directions.

#### 4.3.2 Topological Properties

Topological materials show many novel quantum properties different from the traditional materials,^[^ ^124^ ^]^ and can be divided into three types according to the electron structures, that is, topological insulators,^[^ ^125^ ^]^ topological Weyl semimetal,^[^ ^126^ ^]^ and topological Dirac semimetal.^[^ ^127^ ^]^ Topological Dirac semimetal shows the unique electron structure, in which the energy bands near the Fermi level possess 3D Dirac points, and three adjacent momentum orientations around the Dirac points are all linear dispersion relationships. Such band crossing characteristics are protected by crystal symmetry, which makes them have a very attractive application prospect in electronic devices, semiconductors, and other fields.^[^ ^128^ ^]^ Whereas, topological Dirac materials discovered so far usually contain heavy metal elements, which are confronted with some tough problems that are not conductive to practical applications, for example, environmental pollution, high cost, and material safety. Therefore, it is necessary to explore light elements to compose stable topological materials. Graphene is the typically light Dirac material, whose plenty novel physical phenomenon and electron properties observed by researchers are given by Dirac cones. Nowadays, Dirac materials are no longer limited to carbon materials, they also covers a variety of other materials. ^[^ ^7^ ^]^ As the left side light element of carbon atom, boron has great potential to spawn 2D Dirac materials with similar properties compared with carbon materials in many aspects.

Confirmed by experiments,^[^ ^129^ ^]^ *β*_12_ and *χ*_3_ borophene both host the Dirac cones, while fully hydrogenated borophene, such as *Pmmn* and *Cmmm*, exhibit twisted Dirac cones, with an ultrahigh Fermi speed (3.5 × 10^6^ m s^−1^), about four times than that of graphene (8.2 × 10^5^ m s^−1^);^[^ ^130^, ^131^, ^132^ ^]^ however, both of their Dirac cones are not perfect in shape, leading to the anisotropic transport properties of borophene. The existence of Dirac cones indicates that borophene can exhibit interesting quantum effects, expected to be a pure element material with zero‐mass Dirac–Fermi effect,^[^ ^28^, ^133^ ^]^ and have the prospect in the application of high‐speed, low‐consumption nanoscale electronic devices.

In 2014, Wang et al.^[^ ^28^ ^]^ utilized ab initio evolutionary structure search method and put forward one kind of borophene named *Pmmn* borophene with massless Dirac fermions, which possesses twisted Dirac cone.^[^ ^134^ ^]^ Astonishingly, *Pmmn* is the third metal Dirac material, and the first 2D Dirac boron material. Subsequently, a 2D partially ionic boron sheet named *γ*‐B_28_ is reported by Du et al.,^[^ ^135^ ^]^ which comprises graphene‐like plane and B_2_ pairs, between which there is a strong charge transfer, and exhibits massless double Dirac cones characteristic by a higher Fermi velocity than that in graphene, enhancing the energy stability of P6/mmm borophene.^[^ ^134^ ^]^

**Figure 6:**
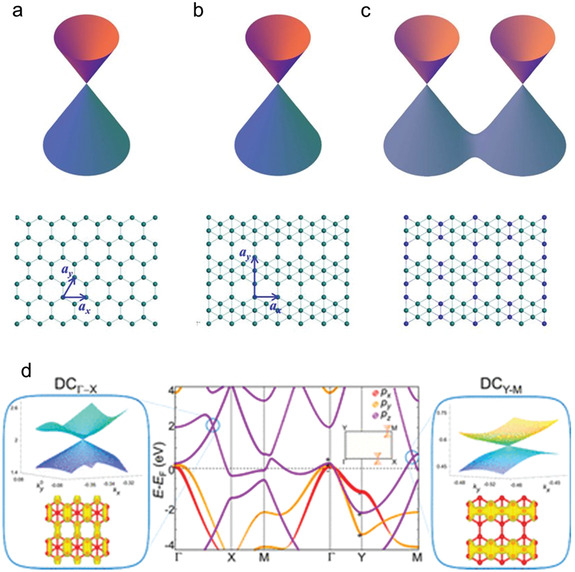
a–c) The Dirac cones and lattices of a) Honeycomb borophene. b) *β*_12_ borophene. c) The *β*_12_ borophene with a 3 × 1 perturbation. a–c) Repoduced with permission.^[^ ^129^ ^]^ Copyright 2017, American Physical Society. d) The band structure of *β*_12_ borophene (centre), and three‐dimensional view of Dirac cone (DC_Γ–X_, left) and Dirac cone (DC_Y–M_, right) with band decomposed isocharge density plot. d) Reproduced with permission.^[^ ^136^ ^]^ Copyright 2018, American Chemical Society

The topological properties of *β*_12_ borophene have been the hotspot as it was successfully synthesized on Ag (111).^[^ ^36^ ^]^ Subsequently, Matsuda et al.^[^ ^129^ ^]^ firstly verified that *β*_12_ borophene possesses Dirac fermions by first principles calculations in 2017, which experimentally confirmed the first non‐honeycomb monolayer Dirac material. It was reported that the *π* bands near the Fermi level of *β*_12_ borophene is separated from *p_z_* orbit, same as in graphene. Moreover, only considering the *p_z_* orbit on the freestanding *β*_12_ borophene, the Dirac cones were revealed in (±2*π*/3a, 0) of the first Brillouin zone (BZ) in the freestanding *β*_12_ borophene through a simple tight‐binding model. Analogue to the honeycomb lattice, *β*_12_ borophene can be disintegrated into two triangular sublattices, and thus host Dirac cones, and the splitting of the Dirac cones are influenced by sublattice symmetry (**Figure** **6**a–c). Meanwhile, Matsuda et al. also proposed that each Dirac cone can be split by utilizing the periodic perturbations, and the split Dirac cones are nonconcentric, different from the graphene. Gupta et al.^[^ ^136^ ^]^ have explored the origin of Dirac fermions in *β*_12_ on Ag (111) and freestanding *β*_12_ borophene, respectively. According to their report, there are one topologically nontrivial Dirac nodal line and two Dirac cones in *β*_12_ borophene, among which two Dirac cones are along the Γ–X (DC_Γ–X_) and Y–M (DC_Y–M_) directions, respectively (Figure 6d). In addition, although the Dirac cones of Ag(111)‐supported *β*_12_ borophene can produce a gap; the nodal line is still topologically protected. The nontrivial topological states in these *β*_12_ borophenes can support further understanding of the topological properties of borophene, and thus be in favor of playing a role in basal research and applications. In 2017, Chen et al.^[^ ^137^ ^]^ predicted a new 2D boron structure named hr‐sB with two types of Dirac fermions corresponding to the Dirac nodal line and strong anisotropic gradient Dirac cones apart, different from the traditional Dirac cones observed in *β*_12_. Inspired by the honeycomb structure in graphene, the research relevant to the Dirac cones of honeycomb in‐plane boron is also intriguing. Furthermore, a honeycomb borophene oxide named *h*‐B_2_O found recently has excellent conductivity as the 2D topological nodal‐loop metal, and thus is an exceedingly promising anode material for batteries with enormous high capacity.^[^ ^138^ ^]^

Nowadays, the studies referring to the topological properties have been not limited to the model of Weyl or Dirac fermions, and the symmetry analysis have also extended to the crystals with magnetic order and interactions.^[^ ^139^ ^]^ Recently, Ezawa et al.^[^ ^140^ ^]^ proposed that there may be triplet fermions in *β*_12_ borophene and constructed three‐band theories for triplet fermions. All above understandings of the topological properties of 2D boron materials lay the solid foundation for subsequent electrical application research.^[^ ^141^ ^]^

#### 4.3.3 Superconductivity

It is reported that the relatively low atomic mass of boron can produce strong electron–phonon coupling, and the metallicity of borophene can make itself produce a higher carrier concentration,^[^ ^142^, ^143^, ^144^ ^]^ both of which are key factors in the formation of conventional superconductors, proving that borophene has the potential to become a superconductor. Remarkably, Yakobson et al.^[^ ^120^ ^]^ predicted its critical temperature to be *T*_c_ ≈ 10–20 K by theoretical calculations, which is much higher than that of graphene (*T*_c_ ≈ 8.1 K in theory^[^ ^145^ ^]^ and 4.5 K experimentally).^[^ ^146^ ^]^ Nevertheless, the electron doping and tensile strain can induce the suppression of superconductivity for borophene,^[^ ^147^, ^148^, ^149^ ^]^ making it difficult for the critical temperature of borophene to be detected in experiments demonstrably. However, once these issues are addressed, these properties will increase the flexibility of design and convenience for borophene in superconducting devices.

#### 4.3.4 Semiconductivity

Scientists have experimentally shown that several phases of borophene also have semiconductivity because of the non‐zero bandgaps.^[^ ^43^, ^150^, ^151^ ^]^ Based on PBEO hybrid calculation results, Zeng et al.^[^ ^43^ ^]^ found that *α*‐ and *α*′‐sheets (slightly buckled *α*‐sheet) are both small‐gap semiconductors with indirect bandgaps of 1.40 and 1.10 eV, respectively, among which the *α*′‐sheet has the greatest cohesive energy and is probably the most stable buckled borophene. Kou et al.^[^ ^151^ ^]^ found that though minimal, the surface bandgap of the thicker borophene can still be changed by altering the applied stress, further causing a change in electron mobility and thereby transforming the conductivity type of borophene from metallic into semiconducting. In this case, a higher electron mobility can be obtained by simply changing the applied stress. Therefore, *γ*‐B_28_ borophene has application prospects in the field of pressure‐sensitive and photosensitive devices.

#### 4.3.5 1D Nearly Free Electron States

Characterized by the effective mass almost in accordance with the free electron mass (*m*_e_),^[^ ^152^, ^153^ ^]^ nearly free electron (NFE) states show typically parabolic energy dispersion, whose electron transport properties are fairly remarkable and can be applied in electron emitters.^[^ ^154^, ^155^ ^]^ The NFE states can widely exist on low‐dimensional material surfaces to which the confinement potential is perpendicular, such as 0D C_60_ molecules,^[^ ^156^, ^157^ ^]^ 1D nanotubes and nanoribbons,^[^ ^158^, ^159^ ^]^ and 2D graphene,^[^ ^160^ ^]^ whereas due to the existence of potential gradient usually normal to the basal plane of material, these NFE states prefer to extend in the vacuum region instead of staying around the basal plane, thus imposing restrictions on their superiority in the transport properties.^[^ ^161^ ^]^

Wu et al.^[^ ^161^ ^]^ have observed delocalized 1D NFE states in an Ag (111) supported borophene homojunction, which consists of two (2,3) domains (i.e., the v_1/6_^[^ ^162^ ^]^ or *β*_12_^[^ ^13^ ^]^ sheet) interconnecting the two (2,2) ribbons as a line defect, using STM combined with first‐principles calculations. It is noteworthy that the NFE states in borophene are quite different from these in other low‐dimensional materials just mentioned above; the former of which are still held near the borophene surface because of the as‐formed in‐plane potential gradient, experiencing less disturbance along the perpendicularity of the plane. This unique mechanism means that the NFE states in borophene may be applied in the field of charge transport. Aiming at this important discovery, Wu et al. attempted to change the NFE state closer toward the Fermi level by deepening the potential well so that this state can influence the electron transport much better. Finally, comprehensive calculations indicate that the optimal position for the NFE state is locating slightly beyond the Fermi level, so as to take part in the electron transport efficiently, by applying an in‐plane strain of 2% vertical to the line defect and thus inducing a deeper potential well. In addition, the NFE states may provide an ideal ballistic transport channel owing to its insensitivity to the transport scattering, which can help to obtain a remarkably longer transport relaxation time to reduce the heat generation and electron resistance.

### 4.4 Thermal Properties

Borophene possesses exceptional thermal properties, including thermal stability and thermal conductivity. In recent years, studies have shown that its thermal properties have a great relationship with the structure of borophene. The thermal stability and thermal conductivity of borophene will be described in detail below.

In recent years, many different types of borophene have been theoretically predicted and experimentally confirmed,^[^ ^13^, ^36^, ^43^ ^]^ including the *β*_12_, *δ*_6_, and *χ*_3_ phases, which were grown epitaxially on Ag(111). Once removed from the substrate, they may be thermally unstable, especially the *δ*_6_ phase. To control the instability of *δ*_6_ phase borophene, Zhou et al.^[^ ^106^ ^]^ developed two efficient empirical potentials for molecular dynamics simulations to figure the interaction between low‐energy triangular structures and borophene: the linear potential valence force field mode and nonlinear Stillinger–Weber potential. In addition, another strategy to eliminate the instability of borophene is to produce fully hydrogenated borophene.^[^ ^163^, ^164^ ^]^ It has been noticed that phonon frequencies in the Brillouin zone are positive from its phonon spectrum,^[^ ^163^ ^]^ which indicates dynamic stability.^[^ ^134^ ^]^

Interestingly, recent studies have shown that borophene exhibits special properties in thermal transport, which vary according to its structure. Generally, when the conductor is in a temperature gradient, thermal transport is the heat flux, while this process has a complex phonon transport and scattering mechanism.^[^ ^134^ ^]^ By non‐equilibrium Green's function (NEGF) and ab initio calculations,^[^ ^29^ ^]^ it was found that borophene shows a superior lattice thermal conductivity in the ballistic regime, exceeding that of graphene. This could result from the light mass, short B–B bonds, unusual phonon transmission, strong structural anisotropy of borophene, resulting in a higher phonon group velocity.^[^ ^29^, ^165^ ^]^ Moreover, in MD simulations, the calculated thermal conductivity exhibits a great size dependence generally, when the specimen length is shorter than its permitted phonon mean free path.^[^ ^166^ ^]^ The thermal conductivity calculated by MD increased with the increase in sample length, which is similar to that calculated by phonon Boltzmann transport equation.^[^ ^134^, ^167^ ^]^ However, when the length is infinite, borophene's thermal conductivity is greatly inhibited because of the buckling of it, which increases the out‐of‐plane phonon scattering and leads to stronger diffusion thermal transport.^[^ ^110^ ^]^ Furthermore, the bulked structure in borophene makes its thermal transport anisotropic.

The phonon frequencies of various borophene phases are close to each other because borophene is composed of the same boron atom. Nonetheless, the quantities of boron atoms in each unit cell is not the same, leading to distinct geometric symmetries and different numbers of optical phonon branches, resulting in multiple thermal transport.^[^ ^43^, ^134^, ^165^ ^]^ For low frequency phonons, similar to graphene, the phonon transmission in borophene is almost isotropic, whereas for high‐frequency phonons, the transmission is one‐dimensional, leading to ultrahigh thermal conductivity.^[^ ^29^ ^]^ This indicates that the different phonon scattering rates give rise to the observed differences in thermal conductivity and thermal properties. For example, *β*_12_ phase borophene is highly anisotropic while the *α* phase is isotropic; *α* phase is intrinsically stable, while the *δ*_6_ phase is thermally unstable.^[^ ^29^, ^30^, ^165^ ^]^

The special thermal properties of borophene described above provide guidance for the likely application of these materials, able to meet the different requirements of different industries. For example, high thermal conductivity can help to eliminate accumulated heat in photovoltaic and electronic equipment, while the thermoelectric and thermal insulation industries require low thermal conductivity materials. The multiplicity of thermal transport in borophene makes its application in thermal management and transparent conductors possible.

## 5 Applications of Borophene

A large amount of theoretical and experimental data indicated that borophene has excellent properties with potential in various applications in many fields such as energy storage,^[^ ^168^ ^]^ electronic devices,^[^ ^169^, ^170^, ^171^ ^]^ and biomedicine,^[^ ^25^ ^]^ among others. The size and physicochemical properties of borophene play a decisive role in its further application in electronic devices and many other fields. However, the large‐scale production of large surface area borophene in the laboratory remains challenging. The currently produced borophene is small in size (nanometer level) and unstable in the free state, representing barriers to the further application of borophene in electronic devices, but showing strength in biomedicine. Nanosized materials are popular in the field of tumor therapy. Since the enhanced permeability and retention (EPR) effect of tumors, drug carriers with sizes ranging from 10 to 1000 nm will be enriched at the tumor site, so as to achieve the passive targeting of tumors and improve the efficacy of drugs and reduce the side effects.^[^ ^172^, ^173^ ^]^ In addition, free borophene is easy to combine with other elements or groups to make itself stable, providing borophene with a great advantage in drug delivery. Intelligent delivery of drugs often requires the modification of special groups on the carrier to achieve accurate release at the tumor site or enhance the stability of the delivery system in the body.^[^ ^174^, ^175^, ^176^ ^]^ The modification of special groups on borophene cannot only endow a boron drug carrier with special functions, but also resolve the challenges of the stability of boron. Here, we review the recent applications of borophene, mainly focusing on emerging biomedical fields.

### 5.1 Supercapacitors

Supercapacitor is a new type of energy storage device, with the remarkable properties of low cost, long cycling life, fast charge/discharge rate, high power density, and low energy density. On the basis of the surface charge‐storage mechanism, the electrical conductivity and surface area of the electrode materials prominently affect the supercapacitor performance.^[^ ^177^, ^178^, ^179^ ^]^ Recent developments in 2D nanomaterials have afforded diversely promising candidates for supercapacitors,^[^ ^180^, ^181^, ^182^, ^183^ ^]^ with ultrahigh electrical mobility, extremely large surface area, and ultrathin thickness.^[^ ^184^, ^185^ ^]^

**Figure 7:**
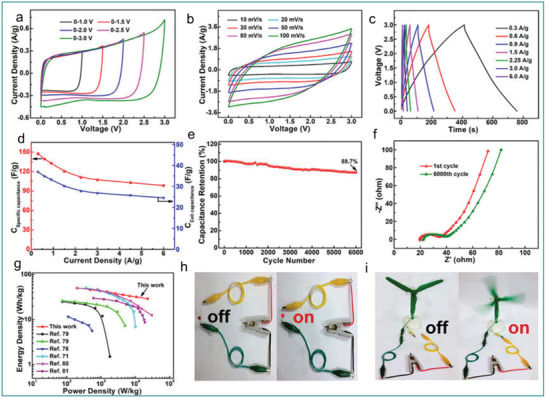
Electrochemical properties of prepared B sheets based supercapacitor. a) Cyclic voltammetry curves obtained under different voltage. b) Cyclic voltammetry curves collected under various scan rates. c) GCD curves tested under different current densities. d) The corresponding cell capacitance (blue curve) and specific capacitance (red curve) under various current densities. e) Cycling stability for 6000 cycles (scan rate: 50 mV s^−1^). f) Nyquist plots were collected at the beginning and end of 6000 cycles. g) Ragone plot in ionic liquid. The prepared battery can be used as a power source for light (h) and fan (i). a–i) Reproduced with permission.^[^ ^86^ ^]^ Copyright 2018, American Chemical Society.

Recent theoretical research has demonstrated that borophene, as a 2D material, exhibits better properties than graphene in some electrochemical aspects, thus being a more hopeful electrode material for batteries and supercapacitors. These properties are described as follows: I) large and stable voltage window: As shown in the **Figure** **7**a, the supercapacitor assembled with DMF‐exfoliated borophene exhibits up to 3.0 V of voltage window; furthermore, Figure 7b shows that even at a scan rate of 100 mV s^−1^, all the cyclic voltammetry curves reveal a nearly rectangle‐like shape, ^[^ ^186^ ^]^ indicating a fast charge/discharge property and a fairly good capacitive behavior (Figure 7c). II) Good specific capacitance: At a current density of 0.3 A g^−1^, a maximum specific capacitance of 147.6 F g^−1^ is achieved (Figure 7d). The maximum specific capacitance is much higher than the supercapacitor based on carbon‐based materials and bulk boron.^[^ ^187^, ^188^ ^]^ III) Excellent rate capability: The current density rises from 0.3–6.0 A g^−1^, about 20‐fold,^[^ ^189^, ^190^ ^]^ the specific capacitance is 98.3 F g^−1^, and the capacitance retention rate is 66.6%.^[^ ^86^ ^]^ IV) Good cycling stability: Figure 7e shows that the initial specific capacitance retention is still 88.7% after 6000 cycles at a scan rate of 50 mV s^−1^, ^[^ ^86^ ^]^which is equal to or even better than that of carbon‐based supercapacitors, even though there is a slight decrease in capacitance after 6000 cycles tested by electrochemical impedance spectroscopy in Figure 7f. V) Excellent energy density: Under 478.5 W kg^−1^ power density, energy density of 46.1 Wh kg^−1^ can be achieved (Figure 7g),^[^ ^191^ ^]^ which is equal to or even higher than supercapacitors made of carbon. In the practical applications, after charging for a few seconds, one coin cell successfully powered light (Figure 7h) or drove fan (Figure 7i). Thus, with the characteristics of low mass density, high specific surface area, remarkable electronic conductivity, etc., the application prospects of borophene nanomaterials for next‐generation photovoltaics^[^ ^184^ ^]^ and energy storage is very promising.

### 5.2 Batteries

The properties of metallicity and low atomic weight make it possible for borophene to be the anode of batteries. Theoretical studies have shown^[^ ^192^ ^]^ that when the intrinsic hollow hexagonal vacancies in borophene tightly hold adsorbed metals like lithium and sodium, the stability of the entire system remains high or even improves. More importantly, borophene has no general problems of serious volume expansion, slow kinetics, and low intercalation utility, all of which exist in the common anode materials such as NiCo_2_O_4_, hard carbon, and Sn.^[^ ^193^, ^194^, ^195^ ^]^ In summary, borophene has the following advantages as the anode, especially for Li‐ and Na‐ion batteries, as theoretically predicted.

#### 5.2.1 Stable Conductive Properties

**Figure 8:**
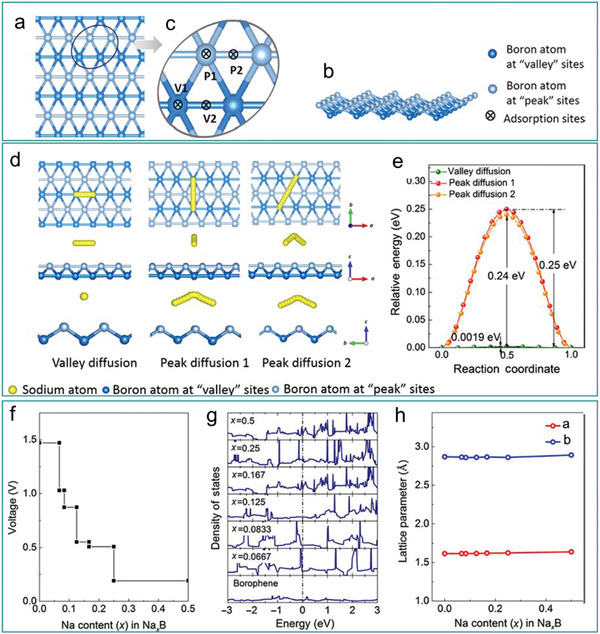
a) Top view, b) side view, and c) the chosen adsorption sites of *Pmmn* borophene. d) Three sodium diffusion routes on borophene. e) The energy curves of diffusion routes. f) Voltage change during the sodiation process. g,h) Borophene's DOS and lattice parameters under various sodium concentrations. a–h) Reproduced with permission.^[^ ^196^ ^]^ Copyright 2016, Elsevier.

*Pmmn* borophene has a unique buckling structure (**Figure** **8**a–c), in which the boron atoms can be divided into two groups, “peak” and “valley,” according to their position heights.^[^ ^36^ ^]^ The layer structure is loose packed, which can ensure a small volume expansion (only 2%) and lattice change after being embedded in by different concentrations of metal atoms like sodium and lithium. Thus, borophene can show good structural integrity and cycle stability as the battery anode material.

#### 5.2.2 Extraordinary Rate Capability

Based on the special structure of borophene, Xu et al.^[^ ^196^ ^]^ obtained an ultralow diffusive energy barrier (0.0019 eV) of sodium in the “valley direction” by DFT calculations, which is much lower than that of traditional anode materials such as Na_2_Ti_3_O_7_ and Na_3_Sb (0.19 and 0.21 eV, respectively).^[^ ^197^, ^198^ ^]^ More surprisingly, the diffusion rate in that direction was more than 1000 times higher than that of Na_2_Ti_3_O_7_ and Na_3_Sb.^[^ ^197^, ^199^ ^]^ Finally, the diffusion energy barrier of Li was slightly above that of Na through the anisotropic diffusion properties (Figure 8d,e).^[^ ^171^ ^]^ All of these observations indicate that borophene has an remarkable rate ability as the anode of Li‐ or Na‐ion batteries, which can obviously solve the problem of slow kinetics.^[^ ^196^ ^]^

#### 5.2.3 Ultrahigh Theoretical Capacity

Taking Na‐ion batteries as an example, when the Na/B (borophene) ratio reaches 0.5 (sodium completely uniformly covers the upper and lower sides of the borophene), the value of the theoretical capacity reaches a maximum (1218 mAh g^−1^ Na_0.5_B),^[^ ^196^ ^]^ which is the maximum value and about three times higher than that of the graphite anode (372 mAh g^−1^). The situation is even better for lithium (Li_0.75_B) because of the larger atomic radius, whose theoretical capacity is 1860 mAh g^−1^, about four times higher than that of graphite anode.^[^ ^171^ ^]^ Thus, borophene can significantly increase the intercalation utility of metals, boosting battery energy densities and power densities.

#### 5.2.4 Effectively Avoiding the Dendrite Formation

Li‐ and Na‐ion batteries are both challenged by dendrite issues because of containing highly reactive metals with a low melting temperature,^[^ ^200^, ^201^ ^]^ presenting a serious safety concern that has not yet been effectively solved. However, borophene may be able to reverse this. Xu et al. ^[^ ^196^ ^]^ found that the calculated average open‐circuit voltage of a Na‐ion battery with borophene as the anode material is as low as 0.53 V during charging and the bond between borophene and Na is strong. (Figure 8f–h). Both exhibit that metal atoms uniformly covered borophene rather than clustering to form a separate metal phase, which can effectively inhibit dendrite formation while maintaining a high energy density.

Although the above data indicate that borophene is a promising battery anode material, the current research remains entirely theoretical, and vast experiments are still needed to confirm its practical applications.

### 5.3 Hydrogen Storage

**Figure 9:**
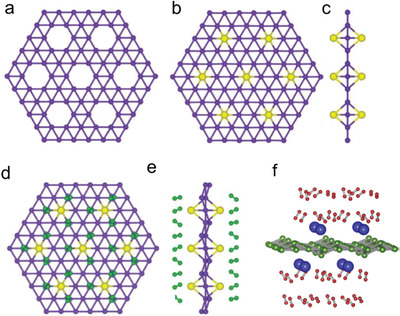
a) Structure of *α*‐sheet borophene without Li. b,c) Optimized structure of *α*‐sheet borophene with Li atoms (yellow spheres). d,e) Optimized structure of Li‐decorated *α*‐sheet borophene after H_2_ adsorption. a–e) Reproduced with permission.^[^ ^209^ ^]^ Copyright 2009, American Chemical Society. f) Optimized structure of Ca‐decorated (blue spheres) *α*‐sheet borophene after H_2_ adsorption. f) Reproduced with permission.^[^ ^206^ ^]^ Copyright 2016, Elsevier.

Considering finite fuel and environmental issues, the development of a material suitable for hydrogen storage is urgent. To assess the hydrogen storage ability of a material, two indices are required, namely adsorption energy and H_2_ adsorption capacity. With regards to adsorption energy, the adsorption energy in the range of 0.2–0.6 eV is requirement to make sure that H_2_ molecules can be adsorbed on the surface of materials stably and easily. With regards to H_2_ adsorption capacity, the target set by the US Department of Energy is of 7.5 wt%. Recently, borophene was found to be a candidate for H_2_ storage with its merits of light weight and large specific surface area, especially when adding with graphene or transition metals.^[^ ^202^, ^203^, ^204^ ^]^ Previous studies have analyzed the adsorption energy of the H_2_ on the surface of pure borophene and found that the maximum is less than 0.01 eV, which is too small for the practical storage of H_2_. Fortunately, with the aid of alkali or transition metals, the chemical activity can be enhanced and the interaction between borophene and H_2_ will be strengthened. During H_2_ adsorption, borophene does not need to generate defects to adsorb metals, because its HHs can be used as metal adsorption sites (**Figure** **9**a–c).^[^ ^39^ ^]^ Tang et al.^[^ ^205^ ^]^ found that *β*_12_ phase borophene with Ca decoration is a perfect candidate for H_2_ storage. The *β*_12_ phase borophene can naturally form HHs, giving Ca a good absorption site (Figure 9f).^[^ ^206^ ^]^ The H_2_ adsorption capacity is about 8.92 wt%. For *χ*_3_ phase borophene with Ca; it can also achieve an H_2_ adsorption capacity of 7.2 wt%,^[^ ^202^ ^]^ closing to graphene.^[^ ^39^, ^207^ ^]^

It has also been proved that, when using lighter metal elements, the H_2_ storage abilities can be greatly improved. For example, Li‐decorated *β*_12_ phase borophene can achieve a H_2_ adsorption capacity of 10.85 wt%^[^ ^208^ ^]^ and *α*‐sheet decorated with Li can adsorb up to a maximum of 10.75 wt% H_2_, representing the capturing of up to 3 H_2_ molecules for each Li atom (Figure 9d,e).^[^ ^209^ ^]^ In addition, Li‐decorated‐buckled triangular borophene, the H_2_ adsorption capacity is 13.7 wt%.^[^ ^39^, ^210^ ^]^ Furthermore, Li‐decorated borophene (*η* = 1/8) with a H_2_ storage capacity of 15.26% has been reported.^[^ ^204^ ^]^ The adsorption energy and H_2_ adsorption capacity of Ca/Li‐decorated borophene are listed in **Table** **3**. All above can sufficiently prove that borophene with Ca/Li decoration possesses prospective potential in H_2_ storage applications.

**Table 3:** Adsorption energy (*E*) and hydrogen adsorption capacity of Ca/Li‐decorated borophene

Metal	Structure	*E* [eV/H_2_]	Capacity [wt%]	Ref.
Ca	*α*‐sheet	0.19	12.68	^[^ ^206^ ^]^
	*β*_12_ sheet	≈0.2	8.92	^[^ ^205^ ^]^
		0.24	9.5	^[^ ^202^ ^]^
	*χ*_3_	0.23	7.2	^[^ ^202^ ^]^
Li	*α*‐sheet	0.15	10.75	^[^ ^209^ ^]^
	*β*_12_ sheet	0.22	10.85	^[^ ^208^ ^]^
	*χ*_3_	0.39	10.79	^[^ ^266^ ^]^
	Borophene (*η* = 1/7)	0.35	9.22	^[^ ^267^ ^]^
	Borophene (*η* = 1/8)	0.23	15.26	^[^ ^204^ ^]^

### 5.4 Bioimaging

Bioimaging, including fluorescence imaging,^[^ ^211^, ^212^ ^]^ photothermal imaging,^[^ ^213^ ^]^ and photoacoustic (PA) imaging,^[^ ^214^ ^]^ can provide the location and size of tumors and is an important technology for the clinical diagnosis and treatment of tumors. The lesion information displayed by each imaging mode is different, and each imaging mode has its own advantages and disadvantages. Therefore, tumor information can be more comprehensively provided through a tumor multimodal imaging platform, which can greatly reduce the risk of misdiagnosis.

**Figure 10:**
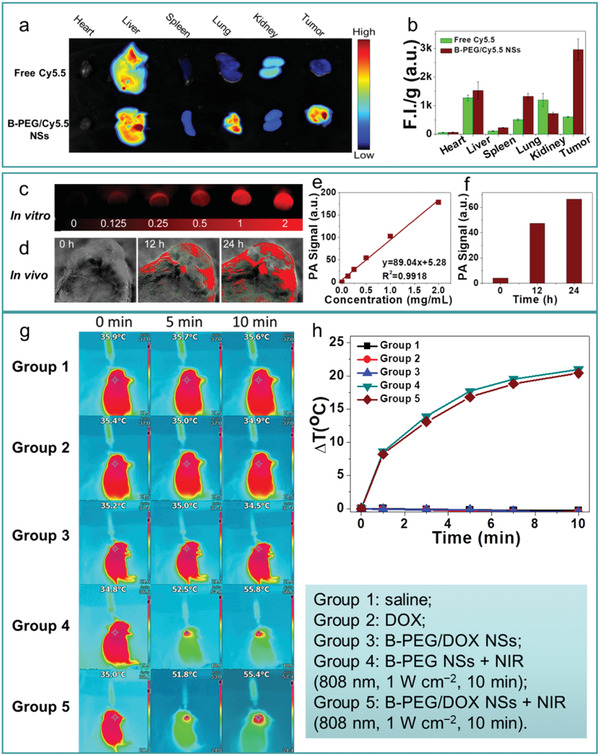
Multimodal imaging of B‐based NSs. a) Detecting major organs and tumors with fluorescence imaging. b) Semiquantitative biodistribution of major organs and tumors. c) PA images of B‐PEG NSs with different concentration (0, 0.125, 0.25, 0.5, 0.1, and 0.2 mg mL^−1^) in vivo. d) Tumor PA images. e) Linear relationship between PA values and concentration of B‐PEG NSs. f) PA values' quantitative analysis. g) Photothermal images and h) temperature profile of mice. a–h) Reproduced with permission.^[^ ^25^ ^]^ Copyright 2018, Wiley‐VCH.

Ji et al.^[^ ^25^ ^]^ constructed a borophene‐based tumor multimodal imaging platform that can simultaneously perform fluorescence imaging, PA imaging, and photothermal imaging. The fluorescent material is loaded onto the carrier and the formulation is enriched at the tumor by active targeting or passive targeting methods. A fluorescence imaging picture of a tumor mouse injected Cy5.5‐labeled borophene modified by PEG (Cy5.5‐labeled B‐PEG NS) (**Figure** **10**a,b) clearly distinguished between malignant tumors and normal tissues. Compared with free fluorescent dye Cy5.5, Cy5.5‐labeled B‐PEG NS has a longer blood circulation time and better tumor accumulation because of the EPR effect. Furthermore, B‐PEG NS is also a potential PA agent. PA imaging is a non‐destructive bioimaging method that uses ultrasound as a medium. Compared to optical imaging and acoustic imaging, PA imaging overcomes many of the shortcomings of these two imaging technologies, and increases the probing depth, image contrast, and spatial resolution. PA imaging can show the tumor structure diagram of tumor mouse at different times following the injection of B‐PEG NS (Figure 10d,f), and the intensity of the PA signal is positively correlated with the concentration of B‐PEG NS (Figure 10c,e)—the higher the B‐PEG NS concentration, the stronger the signal. The above results indicated that B‐PEG NS can be effectively enriched in tumor sites and is a potential PA agent. In addition, borophene has a good photothermal conversion rate and photothermal stability, converting light energy into heat energy under the illumination of near‐infrared (NIR) light. The distribution of borophene and temperature changes in various organs can also be obtained by an infrared thermal camera. For example, in an experiment, mice were divided into five groups, where groups four and five were given NIR irradiation after intravenous injection of B‐PEG NSs or B‐PEG/DOXNSs (B‐PEG NSs loaded doxorubicin) and the temperature at the tumor of these two groups increased from 308 to 328 K, whereas the temperature of other groups without B‐PEG NSs or NIR were maintained stable at ≈308K (Figure 10g,h). In conclusion, borophene can be potentially used in tumor multimodal bioimaging platforms, helping to achieve the accurate diagnosis and precise treatment of tumors.

### 5.5 Drug Delivery

**Figure 11:**
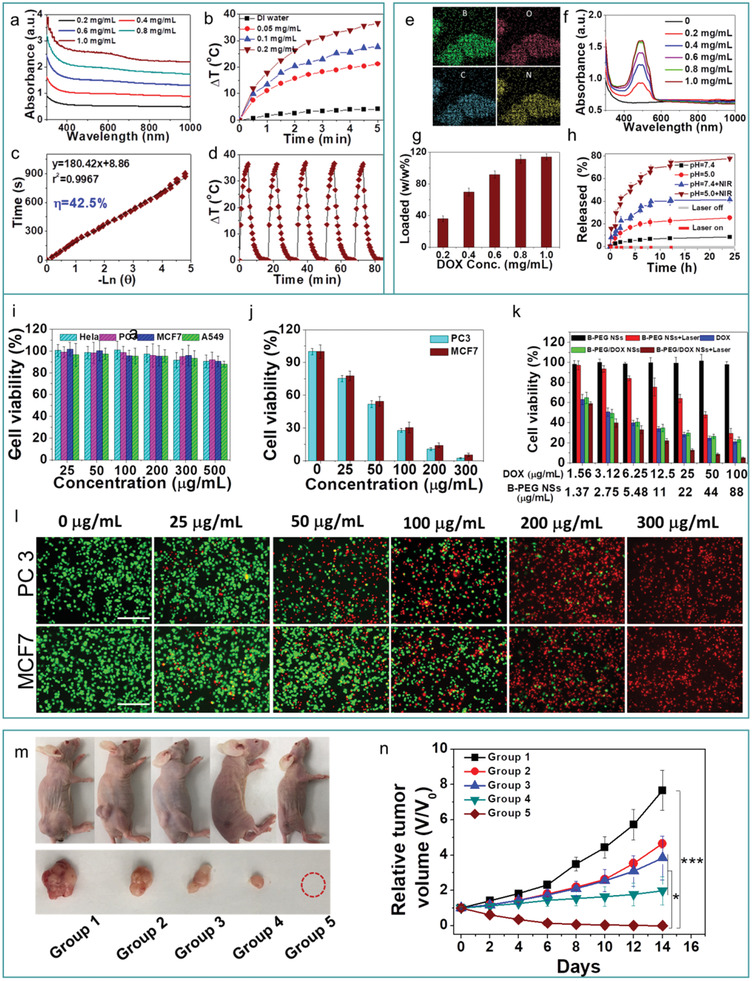
a) UV spectrum of B‐PEG NSs. b) Temperature‐change profile of B‐PEG NSs under 808 nm laser. c) Linear relationship between −ln*θ* and time. d) Heating profile of B‐PEG NSs after five cycles. e) Scanning transmission electron microscopy–EDS images of B‐PEG/DOX NSs. f) UV spectrum of borophene loading with DOX. g) Borophene's ability to load chemotherapy drugs. h) Release curves of DOX under different pHs. i–n) Cell experiments. i) Safety evaluation of borophene in different cells (without laser). j) Toxicity evaluation of borophene in different cells (808 nm laser). k) Relative viability of tumor cells under different treatments. l) CLSM images of tumor cells dyed with PI (dead, red) and AM (live, green). m) Digital photographs of mice and representative tumors in different groups treated for 14 days. n) Body weight of mice. G 1: control; G 2: chemotherapy drugs; G 3: borophene + chemotherapy drugs; G 4: borophene + 808 nm laser; G 5: borophene + chemotherapy drugs + 808 nm laser. a–n) Reproduced with permission.^[^ ^25^ ^]^ Copyright 2018, Wiley‐VCH.

The localized release of chemotherapeutic drugs is the key to reduce the side effects of chemotherapy. Therefore, it is especially important to design a smart tumor microenvironment response drug‐release platform. The ultrahigh specific surface area of borophene provides space for drug loading and anchor points for functional group modification, making it a suitable material for many aspects of anti‐cancer treatment. In a borophene‐based drug delivery system, doxorubicin (DOX) was incubated with borophene (loading rate: 114%) and the system was coated with PEG‐NH_2_ to achieve long‐lasting circulation of chemotherapeutic drugs in the body (**Figure** **11**e–g) demonstrated successful modification of PEG and loading of DOX.^[^ ^25^ ^]^ As a drug carrier, borophene showed good pH and photothermal responsiveness. At pH 7.4 and pH 5.0, the release rates of DOX by borophene were 8.3% and 24%, respectively (Figure 11h). The pH of the tumor microenvironment is lower than that of normal tissue (literature), which allows borophene to achieve targeted release of the drug at the tumor. In addition, borophene is also a drug carrier for NIR response. Under the above conditions, 5 min of NIR illumination was applied to different pH groups, with the drug release rate at pH 7.4 increasing to 41.4% and that at pH 5.0 increasing to 77.6% (Figure 11h). In summary, borophene with high drug loading capacity and pH and infrared dual response, is a promising integrated drug delivery platform for both the diagnosis and treatment of cancer.

### 5.6 Cancer Therapy

Photothermal therapy can achieve non‐invasive/minimally invasive treatment of cancer. The method uses NIR radiation to irradiate tumor tissue and the tumor tissue temperature is increased due to photothermal conversion, resulting in the death of tumor cells.^[^ ^215^ ^]^ An ideal photothermal therapeutic agent should have low toxicity, strong light absorption, a high photothermal conversion efficiency, and photothermal stability in the NIR window (650–950 nm).^[^ ^216^, ^217^ ^]^ Ji et al.^[^ ^25^ ^]^ used borophene in the photothermal therapy of tumors and achieved good therapeutic effects. Three important criteria for an excellent photothermal agent are photothermal conversion efficiency, photothermal stability, and UV absorbance; these properties of borophene were evaluated. The UV–vis–NIR absorbance of borophene is high (Figure 11a). Borophene was dispersed in an aqueous solution and irradiated with 808 nm infrared light (2 W cm^−2^) for 5 min, and the temperature changes of borophene at different concentrations were recorded (Figure 11b). According to the results, under the above conditions, the highest transition temperature (310 K) was obtained at a concentration of 0.2 mg mL^−1^. Compared with other reported photothermal agents, such as black phosphorus quantum dots (28.4%) and Au nanorods (21%), borophene has a higher photothermal conversion efficiency (42.5%) (Figure 11c). In addition, the stability of borophene was evaluated by repeated NIR irradiation. After five rounds of repeated irradiation and cooling, the photothermal properties of the nanosheets hardly changed, indicating that it has good photothermal stability (Figure 11d). Effectiveness and high biocompatibility are important indicators to assess whether a material can be successfully applied in the field of biomedicine; therefore, the cytotoxicity and photothermal availability of borophene have been evaluated both in vitro and in vivo. Different concentrations of borophene were incubated with four cancer cell types for 48 h and the cell viability was evaluated. Negligible cell toxicity was observed (Figure 11i), indicating that borophene has good biocompatibility. On this basis, borophene was used as a photothermal agent to evaluate the antitumor effect of a boron‐based drug delivery system (Figure 11j–l). At a B‐PEG NSs concentration of 200 µg mL^−1^ and under NIR light, more than 80% of MCF7 tumor cells died and almost 90% of PC3 tumor cells died. Further, following the intravenous injection of B‐PGE/DOX and tumor site irradiation with infrared light, the volume of the tumor on mice was significantly reduced and mouse body weight was hardly affected (Figure 11m,n). In summary, borophene is an excellent anti‐tumor treatment platform with a high photothermal conversion efficiency (42.5%), high photothermal stability, high‐efficiency tumor removal ability, and good biocompatibility.

### 5.7 Biosensors

Biosensors can quickly and accurately detect biomarkers such as hormones, proteins, and glucose with highly specificity and sensitivity,^[^ ^218^ ^]^ and are available for blood sample detection, early cancer diagnosis, and clinical analysis, attracting high attention in the field of biomedicine.^[^ ^6^, ^10^ ^]^ 2D nanomaterials have become excellent candidates for biosensors due to their superior electrochemical performance, high surface‐to‐volume ratios,^[^ ^219^ ^]^ more surface‐active sites,^[^ ^220^, ^221^ ^]^ and high electronic mobilities at monolayer thicknesses.^[^ ^10^, ^222^ ^]^

Borophene shows a vast biosensing application potential for gas detection because of its outstanding large‐surface area and superb absorbability to gas molecules.^[^ ^223^ ^]^ As the biosensor, it can monitor not only non‐toxic gases like ethanol (CH_2_OH)^[^ ^224^ ^]^ and ammonia (NH_3_),^[^ ^225^ ^]^ but also highly toxic gases such as formaldehyde (CHOH), CO, and NO^[^ ^225^ ^]^ that even cannot be monitored by graphene and phosphorene gas biosensors, both of which recently have been considered highly sensitive as biosensors. In addition to the fairly strong adsorption strength of highly toxic gases on borophene surface, the reason why borophene is more suitable to be highly toxic gas biosensors is partly enabled by the electronic bandgap, of which other 2D materials such as graphene lack. The electronic bandgap of borophene can rapidly decrease when the gas adsorbs, facilitating plenty of electrons transferring and enhancing electrical conductivity, and thus makes borophene remarkable in applications of highly toxic gas biosensors. ^[^ ^226^ ^]^

HCOH is known as the highly toxic and irritating carcinogen, and exposure to it may cause undesirable reactions such as watery eyes, respiratory irritation and asthma.^[^ ^227^, ^228^, ^229^ ^]^ However, it is widely used in many industrial and medical fields due to its excellent chemical reactivity and high thermal stability.^[^ ^227^ ^]^ Therefore, it is necessary to develop a highly sensitive HCOH biosensor to effectively avoid the adverse effects that HCOH may result in. Ansari et al.^[^ ^230^ ^]^ studied the application potential of B_36_ as the HCOH biosensor by analyzing its consolidated hexagonal hollow structure with DFT computations, and they found that the presence of HCOH can significantly increase the conductivity of B_36_, thus generating electrical signals. Furthermore, the strength of electrical signals can increase along with more adsorbed HCOH molecules, indicating that B_36_ has a highly sensitivity to the concentration (or pressure) of HCOH and will have great prospect as the HCOH biosensor. From the practical utility point of view, this also indicates that borophene may be more outstanding in detecting the concentration of some toxic gases.

Huang et al.^[^ ^225^ ^]^ have analyzed the transportation characteristics of borophene after gas adsorption of CO and NO, respectively, by means of NEGF‐based methods. It was found that the adsorption energies are extremely high, resulting in large amounts of charge transfer on the surface of borophene, which shows that borophene is the excellent candidate for novel gas biosensors once again. CO and NO have shown good prospects in anti‐inflammatory, antibacterial, antitumor, etc.^[^ ^231^ ^]^ However, there are many uncontrolled factors in the gas transport, such as dose control and targeted delivery. Therefore, safe and controllable releasing and real‐time concentration monitoring in vivo are the keys to facilitate the further clinical transformation of CO and NO gas therapy. Now that borophene biosensors are sensitive to the change of concentration of CO and NO, they can be considered to effectively monitor the gas concentration in CO and NO gas therapy in future research. In addition, the hybrid biosensor composed of borophene and hydrogel‐based polymers that have been used in regenerative medicine as adhesive scaffolds, has been boldly assumed and considered as a smart and highly sensitive device, which can convert pH, temperature and other stimuli into optical, electrical and mechanical signals. This biosensor will be supposed to apply in biomedical fields such as blood testing.^[^ ^232^ ^]^

Although borophene biosensor is a distinguished device in theory, especially for gas detection, the reduced stability after gas adsorption and low reusability because of high adsorption energies are both crucial issues that need to be addressed. Hence, to realize the transformation of borophene biosensors in the biomedicine field, more comprehensive research will be required to clarify the cytotoxic effects, durational stability, effects when close contact with biological tissues, etc.^[^ ^233^ ^]^ In addition, borophene is unstable when exposed to air; therefore, it is necessary to take possible external reactions into consideration, such as reactions with metallic appliances^[^ ^234^ ^]^ or smoking metabolites.^[^ ^235^ ^]^

## 6 Conclusions

Borophene has many fascinating properties, including being light weight, having a good mechanical toughness, excellent superconducting properties, and a series of outstanding biomedical properties increasing its application potential in the fields of electronic equipment and biomedicine. Herein, the developmental history of the theoretical structure of borophene was described and the synthesis methods of borophene, and the factors affecting these were summarized and analyzed in detail. The achievement of experimental synthesis of borophene (a 2D material) has been the focus of attention in this field.

## 7 Perspective

Although there remain various challenges, borophene has great application value in advanced composite materials, flexible electronic products, energy storage materials, and biomedicine, making these materials worth further research. Previously, most research had focused on the development and application of borophene in electronic devices, while few studies addressed the development value of these materials in biomedicine. In fact, boron is inextricably linked with our lives. Boron is a necessity for the formation of ribonucleic acid, which is an important building block of life, so boron may be important for the origin of life.^[^ ^236^ ^]^ As early as 1988, it was discovered that boron can improve bone calcification, which is an essential element of animals and plants.^[^ ^237^ ^]^ Furthermore, boron is abundant in nature. Naturally produced borax (Na_2_B_4_O_7_·10H_2_O) has been used as a medicine in ancient China to treat acute tonsillitis, pharyngitis, stomatitis, and gingivitis.^[^ ^238^ ^]^ Nowadays, with its extremely high specific surface area, good biocompatibility, high photothermal conversion efficiency, and good bioimaging effects, borophene has emerged in cancer diagnosis and treatment. Since ancient times, the contribution of boron and its related compounds to human health has been exciting, and there is reason to believe that boron‐related products may make a major difference in biomedical fields.^[^ ^239^ ^]^ The advantages and challenges of borophene in biomedical field will be summarized and forecasted below.

### 7.1 Potential Applications of Borophene in the Field of Biomedicine

It is expected that borophene will be used in many interesting applications in biomedicine, in addition to photothermal therapy, drug delivery, tumor multimodal imaging, and other functions. Tumor vaccines have been the center of attention in recent years.^[^ ^240^, ^241^ ^]^ Compared with chemotherapy and radiotherapy, their side effects are smaller, and they can prevent tumor growth, spread, and recurrence by activating the patients’ own immune system.^[^ ^242^ ^]^ However, the currently developed tumor vaccines have problems such as a difficulty in collecting immune‐related cells, inefficient antigen delivery, or weak tumor killing ability.^[^ ^243^, ^244^, ^245^ ^]^ Ma et al.^[^ ^246^ ^]^ have developed a “one for all” tumor vaccine delivery platform that integrates antigen presenting cell (APC) recruitment/activation, antigen delivery, and cross‐presentation to solve the existing problems of tumor vaccines, using oxidized graphene (2D material) as a chassis. Compared with 0D spherical nanocarriers, 2D oxidized graphene can promote APCs and subsequent antigen cross‐presentation more effectively, and plays a role as an immune‐related cytokine and antigen reservoir. Experimental observations show that, when oxidized graphene enters macrophages, it folds and cleverly wraps the antigen in wrinkles to protect the antigen from the enzymes in the envelope. Moreover, the folding properties of oxidized graphene are also closely related to autophagy, activation of APCs, and efficient antigen cross‐presentation. Furthermore, oxidized graphene as a 2D nanomaterial has a special dimension that can induce a nano‐biointerface effect that is completely distinct from ordinary spherical rod‐like nanomaterials. It can rub against the cell membrane during the process of phagocytosis of macrophages, accelerating the fluidity of the macrophage membrane, and activating the internal signal by stimulating the integrin *α*v*β*8 on the cell membrane, helping it to activate macrophages and promote the rate of macrophage migration. Thus, 2D materials have great potential in assisting immunotherapy. Borophene, as a 2D material, with excellent mechanical toughness and high flexibility, is very likely to have excellent immune function, including cytokine‐like activity, enzyme‐like activity, and agonist activity mirroring that of oxidized graphene, which make it a potential drug delivery platform for immunotherapy.

Hydrogen gas therapy has good anti‐tumor, anti‐oxidation, cerebral ischemia treatment, and liver disease treatment effects. The potential of borophene in hydrogen storage has been described above, lays a good foundation for the application of borophene in hydrogen therapy.^[^ ^247^, ^248^, ^249^, ^250^ ^]^ In 1975, Fife et al.^[^ ^251^ ^]^ reported that breathing high‐pressure hydrogen can treat squamous cell carcinoma of the skin in mice. Hydrogen can alleviate the adverse reactions of radiotherapy and chemotherapy in malignant tumors and has a good synergistic effect with chemotherapeutic drugs, which can significantly prolong the life‐span of tumor‐bearing animals. In normal cells, hydrogen can reduce cell apoptosis. To date, drinking hydrogen‐rich water and breathing air with a higher concentration of hydrogen are two main hydrogen treatment methods, which are limited by high treatment cost, complicated hydrogen production equipment, limited solubility of hydrogen in water, and a low utilization rate of the diseased organ. Therefore, improving the enrichment and utilization of hydrogen at the lesion site is a challenge for the further development of hydrogen therapy. With the hydrogen storage potential and the unique EPR effect, borophene can be used as a drug‐delivery platform integrating hydrogen therapy and multiple therapies to achieve the efficient delivery of hydrogen.

Brain tumors are difficult to treat lesions; boron neutron capture therapy (BNCT) has been shown to have a good effect on these lesions.^[^ ^252^, ^253^ ^]^ The principle of BNCT involves the stable isotope boron‐10 (^10^B) being enriched in tumor cells followed by irradiation with neutron beams to induce nuclear capture and fission reactions, which destroy cancer cells.^[^ ^254^, ^255^ ^]^ Compared with traditional radiotherapy and chemotherapy, BNCT has the following advantages: I) Tumors have a much higher affinity for boron‐containing drugs than normal tissues, leading to tumor‐targeted therapy; III) BNCT has a good killing effect on both oxygen‐rich and hypoxic tumors, avoiding the dependence of radiotherapy on oxygen; III) compared with chemotherapy and radiotherapy, which have a certain dependence on the cell‐growth cycle, the killing effect of BNCT is independent from the cell growth cycle and is effective for tumor cells in the stationary phase. Therefore, BNCT is considered a more mature anti‐tumor radiotherapy method. Nevertheless, an important condition for BNCT technology is the achievement of a high concentration of boron carriers in the tumors, for which the design and selection of boron carriers is critical.^[^ ^256^ ^]^ Furthermore, increasing drug blood–brain barrier penetration is a major issue in the treatment of brain diseases. Encouragingly, research has found that nanosheets with photothermal effects, such as black phosphorus, can effectively promote drug blood–brain barrier penetration through photothermal effects, and promote the treatment of Alzheimer's disease and depression.^[^ ^257^, ^258^, ^259^ ^]^ Borophene, which has good tumor targeting and photothermal effects, has higher boron purity than other boron‐containing compounds, and is therefore likely to become a potential boron carrier in glioma treatment.

In addition, nanoscaled (1–100 nm) materials tend to exhibit properties that differ from those of the same material at the macroscopic scale.^[^ ^260^ ^]^ In this way, 2D nanomaterials can exhibit activity similar to that of enzymes in the human body, yet with improved stability and efficiency than biological enzymes.^[^ ^261^, ^262^ ^]^ These materials, termed nanozymes, have received extensive attention in the fields of disease diagnosis and treatment. For example, Au nanoparticles,^[^ ^263^ ^]^ carbon nanotubes,^[^ ^264^ ^]^ etc., have good peroxidase‐like catalytic activity, and MnO_2_ 2D nanosheets have glucose oxidase activity, able to catalyze the decomposition of glucose into hydrogen peroxide and gluconic acid.^[^ ^265^ ^]^ Thus, MnO_2_ 2D nanosheets can effectively kill tumors by using starvation therapy in combination with other therapies. The above examples led us to speculate that borophene, which also has a 2D structure and is rich in surface active sites, could also possess certain enzymatic activities.

### 7.2 Challenges for the Further Application of Borophene

Large‐scale production of quality controlled borophene is still a major challenge for further applications of borophene. Both the bottom‐up and top‐down approaches have inherent limitations. CDV and PVD are two classical bottom‐up methods for the preparation of borophene, but these two methods are harsh, have a high cost, and produce materials with a small surface area (larger surface area of borophene is more suitable for further application on electronic equipment). Furthermore, the transfer of borophene from the metal substrate is difficult, and environmental contamination may easily occur, both of which make it more difficult to achieve further applications in electronic devices. Although the top‐down preparation method is more economical and simpler, the thickness of borophene is not uniform and it is difficult to achieve a single‐layer atomic thickness. Therefore, developing an efficient method of synthesizing controllable‐quality borophene will help the further application and development of borophene.

## Conflict of Interest

The authors declare no conflict of interest.

## References

[1] X. Huang , X. Qi , F. Boey , H. Zhang , Chem. Soc. Rev. 2012, 41, 666. 21796314 10.1039/c1cs15078b

[2] X. Huang , Z. Yin , S. Wu , X. Qi , Q. He , Q. Zhang , Q. Yan , F. Boey , H. Zhang , Small 2011, 7, 1876. 21630440 10.1002/smll.201002009

[3] Q. Lu , Y. Yu , Q. Ma , B. Chen , H. Zhang , Adv. Mater. 2016, 28, 1917. 26676800 10.1002/adma.201503270

[4] M. Chhowalla , H. S. Shin , G. Eda , L. J. Li , K. P. Loh , H. Zhang , Nat. Chem. 2013, 5, 263. 23511414 10.1038/nchem.1589

[5] Z. Sun , Q. Fan , M. Zhang , S. Liu , H. Tao , J. Texter , Adv. Sci. 2019, 6, 1901084. 10.1002/advs.201901084 PMC6760473 31572648

[6] K. Ariga , X. Jia , J. Song , J. P. Hill , D. T. Leong , Y. Jia , J. Li , Angew. Chem., Int. Ed. 2020, 59, 15424. 10.1002/anie.202000802 32170796

[7] K. S. Novoselov , A. K. Geim , S. V. Morozov , D. Jiang , Y. Zhang , S. V. Dubonos , I. V. Grigorieva , A. A. Firsov , Science 2004, 306, 666. 15499015 10.1126/science.1102896

[8] A. K. Geim , K. S. Novoselov , Nat. Mater. 2007, 6, 183. 17330084 10.1038/nmat1849

[9] X. Ding , F. Peng , J. Zhou , W. Gong , G. Slaven , K. P. Loh , C. T. Lim , D. T. Leong , Nat. Commun. 2019, 10, 41. 30604777 10.1038/s41467-018-07835-1 PMC6318297

[10] B. L. Li , J. Wang , Z. F. Gao , H. Shi , H. L. Zou , K. Ariga , D. T. Leong , Mater. Horiz. 2019, 6, 563.

[11] C. R. Dean , A. F. Young , I. Meric , C. Lee , L. Wang , S. Sorgenfrei , K. Watanabe , T. Taniguchi , P. Kim , K. L. Shepard , J. Hone , Nat. Nanotechnol. 2010, 5, 722. 20729834 10.1038/nnano.2010.172

[12] S. W. Cao , J. X. Low , J. G. Yu , M. Jaroniec , Adv. Mater. 2015, 27, 2150. 25704586 10.1002/adma.201500033

[13] B. Feng , J. Zhang , Q. Zhong , W. Li , S. Li , H. Li , P. Cheng , S. Meng , L. Chen , K. Wu , Nat. Chem. 2016, 8, 563. 27219700 10.1038/nchem.2491

[14] B. J. Feng , Z. J. Ding , S. Meng , Y. G. Yao , X. Y. He , P. Cheng , L. Chen , K. H. Wu , Nano Lett. 2012, 12, 3507. 22658061 10.1021/nl301047g

[15] L. Chen , C. C. Liu , B. J. Feng , X. Y. He , P. Cheng , Z. J. Ding , S. Meng , Y. G. Yao , K. H. Wu , Phys. Rev. Lett. 2012, 109, 056804. 23006197 10.1103/PhysRevLett.109.056804

[16] Y. Du , J. C. Zhuang , H. S. Liu , X. Xu , S. Eilers , K. H. Wu , P. Cheng , J. J. Zhao , X. D. Pi , K. W. See , G. Peleckis , X. L. Wang , S. X. Dou , ACS Nano 2014, 8, 10019. 25248135 10.1021/nn504451t

[17] J. Gou , L. J. Kong , H. Li , Q. Zhong , W. B. Li , P. Cheng , L. Chen , K. H. Wu , Phys. Rev. Mater. 2017, 1, 054004.

[18] F. F. Zhu , W. J. Chen , Y. Xu , C. L. Gao , D. D. Guan , C. H. Liu , D. Qian , S. C. Zhang , J. F. Jia , Nat. Mater. 2015, 14, 1020. 26237127 10.1038/nmat4384

[19] Z. Y. Ni , Q. H. Liu , K. C. Tang , J. X. Zheng , J. Zhou , R. Qin , Z. X. Gao , D. P. Yu , J. Lu , Nano Lett. 2012, 12, 113. 22050667 10.1021/nl203065e

[20] J. D. Shao , H. H. Xie , H. Huang , Z. B. Li , Z. B. Sun , Y. H. Xu , Q. L. Xiao , X. F. Yu , Y. T. Zhao , H. Zhang , H. Y. Wang , P. K. Chu , Nat. Commun. 2016, 7, 13. 10.1038/ncomms12967 PMC5056460 27686999

[21] W. Tao , X. Zhu , X. Yu , X. Zeng , Q. Xiao , X. Zhang , X. Ji , X. Wang , J. Shi , H. Zhang , L. Mei , Adv. Mater. 2017, 29, 1603276. 10.1002/adma.201603276 PMC5205548 27797119

[22] B. L. Li , R. Li , H. L. Zou , K. Ariga , N. B. Li , D. T. Leong , Mater. Horiz. 2020, 7, 455.

[23] Z. Q. Shi , Q. Q. Li , L. Mei , Chin. Chem. Lett. 2020, 31, 1345.

[24] Z. Shi , Y. Zhou , T. Fan , Y. Lin , H. Zhang , L. Mei , Smart Mater. Med. 2020, 1, 32.

[25] X. Ji , N. Kong , J. Wang , W. Li , Y. Xiao , S. T. Gan , Y. Zhang , Y. Li , X. Song , Q. Xiong , S. Shi , Z. Li , W. Tao , H. Zhang , L. Mei , J. Shi , Adv. Mater. 2018, 30, 1803031. 10.1002/adma.201803031 PMC6338531 30019786

[26] W. Tao , X. B. Zhu , X. H. Yu , X. W. Zeng , Q. L. Xiao , X. D. Zhang , X. Y. Ji , X. S. Wang , J. J. Shi , H. Zhang , L. Mei , Adv. Mater. 2017, 29, 1603276. 10.1002/adma.201603276 PMC5205548 27797119

[27] L. M. Zhang , J. G. Xia , Q. H. Zhao , L. W. Liu , Z. J. Zhang , Small 2010, 6, 537.20033930

[28] X.‐F. Zhou , X. Dong , A. R. Oganov , Q. Zhu , Y. Tian , H.‐T. Wang , Phys. Rev. Lett. 2014, 112, 085502.

[29] H. Zhou , Y. Cai , G. Zhang , Y.‐W. Zhang , npj 2D Mater. Appl. 2017, 1, 14.

[30] H. Xiao , W. Cao , T. Ouyang , S. Guo , C. He , J. Zhong , Sci. Rep. 2017, 7, 45986. 28374853 10.1038/srep45986 PMC5379675

[31] G. Forte , A. La Magna , I. Deretzis , R. Pucci , Nanoscale Res. Lett. 2009, 5, 158. 20652134 10.1007/s11671-009-9458-8 PMC2893925

[32] Y. Huang , S. N. Shirodkar , B. I. Yakobson , J. Am. Chem. Soc. 2017, 139, 17181. 29088913 10.1021/jacs.7b10329

[33] X. Liu , Z. Zhang , L. Wang , B. I. Yakobson , M. C. Hersam , Nat. Mater. 2018, 17, 783. 30013053 10.1038/s41563-018-0134-1

[34] X. Liu , M. C. Hersam , Sci. Adv. 2019, 5, eaax6444. 31646179 10.1126/sciadv.aax6444 PMC6788864

[35] I. Boustani , Int. J. Quantum. Chem 1994, 52, 1081.

[36] A. J. Mannix , X.‐F. Zhou , B. Kiraly , J. D. Wood , D. Alducin , B. D. Myers , X. Liu , B. L. Fisher , U. Santiago , J. R. Guest , M. J. Yacaman , A. Ponce , A. R. Oganov , M. C. Hersam , N. P. Guisinger , Science 2015, 350, 1513. 26680195 10.1126/science.aad1080 PMC4922135

[37] Z. Zhang , E. S. Penev , B. I. Yakobson , Chem. Soc. Rev. 2017, 46, 6746. 29085946 10.1039/c7cs00261k

[38] A. J. Mannix , Z. Zhang , N. P. Guisinger , B. I. Yakobson , M. C. Hersam , Nat. Nanotechnol. 2018, 13, 444. 29875501 10.1038/s41565-018-0157-4

[39] J. Shang , Y. Ma , Y. Gu , L. Kou , Phys. Chem. Chem. Phys. 2018, 20, 28964. 30426985 10.1039/c8cp04850a

[40] B. Albert , H. Hillebrecht , Angew. Chem., Int. Ed. 2009, 48, 8640. 10.1002/anie.200903246 19830749

[41] J. Kunstmann , A. Quandt , Phys. Rev. B. 2006, 74, 035413.

[42] Z. A. Piazza , H. Hu , W. Li , Y. Zhao , J. Li , L. Wang , Nat. Commun. 2014, 5, 3113. 24445427 10.1038/ncomms4113

[43] X. Wu , J. Dai , Y. Zhao , Z. Zhuo , J. Yang , X. C. Zeng , ACS Nano 2012, 6, 7443. 22816319 10.1021/nn302696v

[44] V. Bezugly , J. Kunstmann , B. Grundkötter‐Stock , T. Frauenheim , T. Niehaus , G. Cuniberti , ACS Nano 2011, 5, 4997. 21528877 10.1021/nn201099a

[45] X. Yang , D. Yi , J. Ni , Phys. Rev. B. 2008, 77, 41402.

[46] W. J. Tian , B. Hui , H. G. Lu , Y. B. Wu , S. D. Li , J. Cluster Sci. 2013, 24, 1127.

[47] E. S. Penev , S. Bhowmick , A. Sadrzadeh , B. I. Yakobson , Nano Lett. 2012, 12, 2441. 22494396 10.1021/nl3004754

[48] H. Tang , S. Ismail‐Beigi , Phys. Rev. B. 2010, 82, 115412.

[49] K. C. Lau , R. Pandey , J. Phys. Chem. B 2008, 112, 10217. 18661933 10.1021/jp8052357

[50] K. C. Lau , R. Pandey , J. Phys. Chem. C 2007, 111, 2906.

[51] H. Tang , S. Ismail‐Beigi , Phys. Rev. B. 2009, 80, 134113.

[52] H. Tang , S. Ismail‐Beigi , Phys. Rev. Lett. 2007, 99, 115501. 17930448 10.1103/PhysRevLett.99.115501

[53] X. Yu , L. Li , X.‐W. Xu , C.‐C. Tang , J. Phys. Chem. C 2012, 116, 20075.

[54] R. R. Zope , T. Baruah , Chem. Phys. Lett. 2011, 501, 193.

[55] C. Park , J. E. Park , H. C. Choi , Acc. Chem. Res. 2014, 47, 2353. 24901373 10.1021/ar5000874

[56] Y. S. Zhao , P. Zhan , J. Kim , C. Sun , J. Huang , ACS Nano 2010, 4, 1630. 20143788 10.1021/nn901567z

[57] Y. S. Zhao , J. Wu , J. Huang , J. Am. Chem. Soc. 2009, 131, 3158. 19256563 10.1021/ja809360v

[58] Y. Zhang , L. Zhang , C. Zhou , Acc. Chem. Res. 2013, 46, 2329. 23480816 10.1021/ar300203n

[59] O. Akhavan , E. Ghaderi , ACS Nano 2010, 4, 5731. 20925398 10.1021/nn101390x

[60] Y. Chen , X. Wang , C. Yu , J. Ding , C. Deng , H. Zhu , Sci. Rep. 2019, 9, 16338. 31704975 10.1038/s41598-019-52788-0 PMC6841957

[61] J. Yu , J. Li , W. Zhang , H. Chang , Chem. Sci. 2015, 6, 6705. 29861920 10.1039/c5sc01941a PMC5950838

[62] A. Reina , X. Jia , J. Ho , D. Nezich , H. Son , V. Bulovic , M. S. Dresselhaus , J. Kong , Nano Lett. 2009, 9, 30. 19046078 10.1021/nl801827v

[63] X. Li , W. Cai , J. An , S. Kim , J. Nah , D. Yang , R. Piner , A. Velamakanni , I. Jung , E. Tutuc , S. K. Banerjee , L. Colombo , R. S. Ruoff , Science 2009, 324, 1312. 19423775 10.1126/science.1171245

[64] Q. Ji , Y. Zhang , Y. Zhang , Z. Liu , Chem. Soc. Rev. 2015, 44, 2587. 25256261 10.1039/c4cs00258j

[65] S. Bae , H. Kim , Y. Lee , X. Xu , J. S. Park , Y. Zheng , J. Balakrishnan , T. Lei , H. R. Kim , Y. I. Song , Y. J. Kim , K. S. Kim , B. Ozyilmaz , J. H. Ahn , B. H. Hong , S. Iijima , Nat. Nanotechnol. 2010, 5, 574. 20562870 10.1038/nnano.2010.132

[66] J. H. Han , S. Lee , J. Cheon , Chem. Soc. Rev. 2013, 42, 2581. 23212120 10.1039/c2cs35386e

[67] C. Tan , H. Zhang , Nat. Commun. 2015, 6, 7873. 26303763 10.1038/ncomms8873 PMC4560752

[68] L. Cheng , C. Yuan , S. Shen , X. Yi , H. Gong , K. Yang , Z. Liu , ACS Nano 2015, 9, 11090. 26445029 10.1021/acsnano.5b04606

[69] Y. Wu , B. Yuan , M. Li , W. H. Zhang , Y. Liu , C. Li , Chem. Sci. 2015, 6, 1873. 29308137 10.1039/c4sc03229b PMC5649566

[70] A. J. Mannix , B. Kiraly , M. C. Hersam , N. P. Guisinger , Nat. Rev. Chem. 2017, 1, 0014.

[71] H. Li , J. Wu , Z. Yin , H. Zhang , Acc. Chem. Res. 2014, 47, 1067. 24697842 10.1021/ar4002312

[72] M. Yi , Z. Shen , J. Mater. Chem. A 2015, 3, 11700.

[73] V. Nicolosi , M. Chhowalla , M. G. Kanatzidis , M. S. Strano , J. N. Coleman , Science 2013, 340, 1226419.

[74] L. Yuwen , H. Yu , X. Yang , J. Zhou , Q. Zhang , Y. Zhang , Z. Luo , S. Su , L. Wang , Chem. Commun. 2016, 52, 529. 10.1039/c5cc07301d 26535783

[75] Z. Zeng , Z. Yin , X. Huang , H. Li , Q. He , G. Lu , F. Boey , H. Zhang , Angew. Chem., Int. Ed. 2011, 50, 11093. 10.1002/anie.201106004 22021163

[76] J. Zheng , H. Zhang , S. Dong , Y. Liu , C. T. Nai , H. S. Shin , H. Y. Jeong , B. Liu , K. P. Loh , Nat. Commun. 2014, 5, 2995. 24384979 10.1038/ncomms3995

[77] Z. Zeng , T. Sun , J. Zhu , X. Huang , Z. Yin , G. Lu , Z. Fan , Q. Yan , H. H. Hng , H. Zhang , Angew. Chem., Int. Ed. 2012, 51, 9052. 10.1002/anie.201204208 22887481

[78] B. Anasori , Y. Xie , M. Beidaghi , J. Lu , B. C. Hosler , L. Hultman , P. R. Kent , Y. Gogotsi , M. W. Barsoum , ACS Nano 2015, 9, 9507. 26208121 10.1021/acsnano.5b03591

[79] M. Naguib , J. Halim , J. Lu , K. M. Cook , L. Hultman , Y. Gogotsi , M. W. Barsoum , J. Am. Chem. Soc. 2013, 135, 15966. 24144164 10.1021/ja405735d

[80] M. Naguib , M. Kurtoglu , V. Presser , J. Lu , J. Niu , M. Heon , L. Hultman , Y. Gogotsi , M. W. Barsoum , Adv. Mater. 2011, 23, 4248. 21861270 10.1002/adma.201102306

[81] Q. Zhong , L. Kong , J. Gou , W. Li , S. Sheng , S. Yang , P. Cheng , H. Li , K. Wu , L. Chen , Phys. Rev. Mater. 2017, 1, 021001.

[82] G. Tai , T. Hu , Y. Zhou , X. Wang , J. Kong , T. Zeng , Y. You , Q. Wang , Angew. Chem. Int. Ed. Engl. 2015, 54, 15473. 26510179 10.1002/anie.201509285

[83] R. Wu , I. K. Drozdov , S. Eltinge , P. Zahl , S. Ismail‐Beigi , I. Božović , A. Gozar , Nat. Nanotechnol. 2019, 14, 44. 30510278 10.1038/s41565-018-0317-6

[84] W. Li , L. Kong , C. Chen , J. Gou , S. Sheng , W. Zhang , H. Li , L. Chen , P. Cheng , K. Wu , Sci. Bull. 2018, 63, 282. 10.1016/j.scib.2018.02.006 36658797

[85] B. Kiraly , X. Liu , L. Wang , Z. Zhang , A. J. Mannix , B. L. Fisher , B. I. Yakobson , M. C. Hersam , N. P. Guisinger , ACS Nano 2019, 13, 3816. 30844248 10.1021/acsnano.8b09339

[86] H. Li , L. Jing , W. Liu , J. Lin , R. Y. Tay , S. H. Tsang , E. H. T. Teo , ACS Nano 2018, 12, 1262. 29378394 10.1021/acsnano.7b07444

[87] Y. Liu , E. S. Penev , B. I. Yakobson , Angew. Chem., Int. Ed. 2013, 52, 3156. 10.1002/anie.201207972 23355180

[88] Z. A. Piazza , W. L. Li , C. Romanescu , A. P. Sergeeva , L. S. Wang , A. I. Boldyrev , J. Chem. Phys. 2012, 136, 104310. 22423841 10.1063/1.3692967

[89] A. P. Sergeeva , Z. A. Piazza , C. Romanescu , W. L. Li , A. I. Boldyrev , L. S. Wang , J. Am. Chem. Soc. 2012, 134, 18065. 23030415 10.1021/ja307605t

[90] B. Kiran , S. Bulusu , H. J. Zhai , S. Yoo , X. C. Zeng , L. S. Wang , Proc. Natl. Acad. Sci. USA 2005, 102, 961. 15644450 10.1073/pnas.0408132102 PMC545846

[91] E. Oger , N. R. Crawford , R. Kelting , P. Weis , M. M. Kappes , R. Ahlrichs , Angew. Chem., Int. Ed. 2007, 46, 8503. 10.1002/anie.200701915 17907255

[92] X. Li , W. Cai , L. Colombo , R. S. Ruoff , Nano Lett. 2009, 9, 4268. 19711970 10.1021/nl902515k

[93] P. Vajeeston , P. Ravindran , C. Ravi , R. Asokamani , Phys. Rev. B. 2001, 63, 045115.

[94] J. K. Burdett , E. Canadell , G. J. Miller , J. Am. Chem. Soc. 1986, 108, 6561.

[95] Z. Zhang , Y. Yang , G. Gao , B. I. Yakobson , Angew. Chem. 2015, 54, 13022. 26331848 10.1002/anie.201505425

[96] R. S. Hyam , K. M. Subhedar , S. H. Pawar , Colloids Surf., A 2008, 315, 61.

[97] C. Cepek , R. Macovez , M. Sancrotti , L. Petaccia , R. Larciprete , S. Lizzit , A. Goldoni , Appl. Phys. Lett. 2004, 85, 976.

[98] L. Z. Zhang , Q. B. Yan , S. X. Du , G. Su , H. J. Gao , J. Phys. Chem. C 2012, 116, 18202.

[99] S. Xu , Y. Zhao , J. Liao , X. Yang , H. Xu , Nano Res. 2016, 9, 2616.

[100] H. Liu , J. Gao , J. Zhao , Sci. Rep. 2013, 3, 3238. 24241341 10.1038/srep03238 PMC3831238

[101] J. N. Coleman , M. Lotya , A. O'Neill , S. D. Bergin , P. J. King , U. Khan , K. Young , A. Gaucher , S. De , R. J. Smith , I. V. Shvets , S. K. Arora , G. Stanton , H.‐Y. Kim , K. Lee , G. T. Kim , G. S. Duesberg , T. Hallam , J. J. Boland , J. J. Wang , J. F. Donegan , J. C. Grunlan , G. Moriarty , A. Shmeliov , R. J. Nicholls , J. M. Perkins , E. M. Grieveson , K. Theuwissen , D. W. McComb , P. D. Nellist , V. Nicolosi , Science 2011, 331, 568. 21292974 10.1126/science.1194975

[102] P. Ranjan , T. K. Sahu , R. Bhushan , S. S. Yamijala , D. J. Late , P. Kumar , A. Vinu , Adv. Mater. 2019, 31, 1900353. 10.1002/adma.201900353 31044470

[103] H. Zhai , Y. Zhao , W. Li , Q. Chen , H. Bai , H. Hu , Z. A. Piazza , W. Tian , H. Lu , Y. Wu , Y. Mu , G. Wei , Z. Liu , J. Li , S. Li , L. Wang , Nat. Chem. 2014, 6, 727. 25054944 10.1038/nchem.1999

[104] M. Quy Le , B. Mortazavi , T. Rabczuk , Nanotechnology 2016, 27, 445709. 27678335 10.1088/0957-4484/27/44/445709

[105] B. Mortazavi , O. Rahaman , A. Dianat , T. Rabczuk , Phys. Chem. Chem. Phys. 2016, 18, 27405. 27711458 10.1039/c6cp03828j

[106] Y. Zhou , J. Jiang , Sci. Rep. 2017, 7, 45516. 28349983 10.1038/srep45516 PMC5368563

[107] Z. Zhang , Y. Yang , E. Penev , B. I. Yakobson , Adv. Funct. Mater. 2016, 27, 1605059.

[108] M. Schabel , J. L. Martins , Phys. Rev. B. 1992, 46, 7185. 10.1103/physrevb.46.7185 10002428

[109] H. Wang , Q. Li , Y. Gao , F. Miao , X. Zhou , X. G. Wan , New. J. Phys. 2016, 18, 073016.

[110] B. Liu , K. Zhou , Prog. Mater Sci. 2019, 100, 99. 10.1016/j.pmatsci.2018.07.005 PMC6295417 30568319

[111] Y. Zhao , S. Zeng , J. Ni , Appl. Phys. Lett. 2016, 108, 242601.

[112] T. Li , Phys. Rev. B. 2012, 85, 235407.

[113] X. Zou , Y. Liu , B. I. Yakobson , Nano Lett. 2013, 13, 253. 23227928 10.1021/nl3040042

[114] Q. Wei , X. Peng , Appl. Phys. Lett. 2014, 104, 251915.

[115] H. Zhong , K. Huang , G. Yu , S. Yuan , Phys. Rev. B. 2018, 98, 054104.

[116] I. Boustani , A. Quandt , E. Hernandez , A. Rubio , J. Chem. Phys. 1999, 110, 3176.

[117] M. Evans , J. Joannopoulos , S. Pantelides , Phys. Rev. B 2005, 72, 045434. 10.1103/PhysRevLett.95.106802 16196951

[118] I. Boustani , A. Rubio , J. A. Alonso , Chem. Phys. Lett. 1999, 311, 21.

[119] I. Boustani , Surf. Sci. 1997, 370, 355.

[120] E. S. Penev , A. Kutana , B. I. Yakobson , Nano Lett. 2016, 16, 2522. 27003635 10.1021/acs.nanolett.6b00070

[121] M. I. Eremets , V. V. Struzhkin , H.‐k. Mao , R. J. Hemley , Science 2001, 293, 272. 11452118 10.1126/science.1062286

[122] B. Feng , J. Zhang , R.‐Y. Liu , T. Iimori , C. Lian , H. Li , L. Chen , K. Wu , S. Meng , F. Komori , I. Matsuda , Phys. Rev. B. 2016, 94, 041408.

[123] J. E. Padilha , R. H. Miwa , A. Fazzio , Phys. Chem. Chem. Phys. 2016, 18, 25491. 27711511 10.1039/c6cp05092a

[124] X. L. Qi , S. C. Zhang , Rev. Mod. Phys. 2011, 83, 1057.

[125] T. H. Hsieh , H. Lin , J. Liu , W. Duan , A. Bansil , L. Fu , Nat. Commun. 2013, 4, 1901.

[126] S. Y. Xu , I. Belopolski , N. Alidoust , M. Neupane , G. Bian , C. L. Zhang , R. Sankar , G. Q. Chang , Z. J. Yuan , C. C. Lee , S. M. Huang , H. Zheng , J. Ma , D. S. Sanchez , B. K. Wang , A. Bansil , F. C. Chou , P. P. Shibayev , H. Lin , S. Jia , M. Z. Hasan , Science 2015, 349, 613. 26184916 10.1126/science.aaa9297

[127] X. G. Wan , A. M. Turner , A. Vishwanath , S. Y. Savrasov , Phys. Rev. B. 2011, 83, 9.

[128] D. Zhao , L. Cui , J. Cai , Y. Guo , X. Cui , T. Song , Z. Liu , Phys. Status. Solidi RRL 2020, 14, 1900670.

[129] B. Feng , O. Sugino , R.‐Y. Liu , J. Zhang , R. Yukawa , M. Kawamura , T. Iimori , H. Kim , Y. Hasegawa , H. Li , L. Chen , K. Wu , H. Kumigashira , F. Komori , T.‐C. Chiang , S. Meng , I. Matsuda , Phys. Rev. Lett. 2017, 118, 096401. 28306312 10.1103/PhysRevLett.118.096401

[130] L. Xu , A. Du , L. Kou , Phys. Chem. Chem. Phys. 2016, 18, 27284. 27711580 10.1039/c6cp05405f

[131] C. Hwang , D. A. Siegel , S.‐K. Mo , W. Regan , A. Ismach , Y. Zhang , A. Zettl , A. Lanzara , Sci. Rep. 2012, 2, 590.

[132] P. E. Trevisanutto , C. Giorgetti , L. Reining , M. Ladisa , V. Olevano , Phys. Rev. Lett. 2008, 101, 226405. 19113496 10.1103/PhysRevLett.101.226405

[133] D. Malko , C. Neiss , F. Viñes , A. Görling , Phys. Rev. Lett. 2012, 108, 086804. 22463556 10.1103/PhysRevLett.108.086804

[134] D. Li , Y. Chen , J. He , Q. Tang , C. Zhong , G. Ding , Chin. Phys. B 2018, 27, 1674.

[135] F. Ma , Y. Jiao , G. Gao , Y. Gu , A. Bilic , Z. Chen , A. Du , Nano Lett. 2016, 16, 3022. 27050491 10.1021/acs.nanolett.5b05292

[136] S. Gupta , A. Kutana , B. I. Yakobson , J. Phys. Chem. Lett. 2018, 9, 2757. 29741094 10.1021/acs.jpclett.8b00640

[137] H. Zhang , Y. Xie , Z. Zhang , C. Zhong , Y. Li , Z. Chen , Y. Chen , J. Phys. Chem. Lett. 2017, 8, 1707. 28359148 10.1021/acs.jpclett.7b00452

[138] J. Hu , C. Zhong , W. Wu , N. Liu , Y. Liu , S. A. Yang , C. Ouyang , J. Phys.: Condens. Matter 2020, 32, 065001. 31631885 10.1088/1361-648X/ab4f4d

[139] B. Bradlyn , J. Cano , Z. Wang , M. G. Vergniory , C. Felser , R. J. Cava , B. A. Bernevig , Science 2016, 353, aaf5037. 27445310 10.1126/science.aaf5037

[140] M. Ezawa , Phys. Rev. B. 2017, 96, 035425.

[141] X. Fan , D. Ma , B. Fu , C.‐C. Liu , Y. Yao , Phys. Rev. B. 2018, 98, 195437.

[142] J. Kortus , I. I. Mazin , K. D. Belashchenko , V. P. Antropov , L. L. Boyer , Phys. Rev. Lett. 2001, 86, 4656. 11384307 10.1103/PhysRevLett.86.4656

[143] H. J. Choi , D. Roundy , H. Sun , M. L. Cohen , S. G. Louie , Nature 2002, 418, 758. 12181561 10.1038/nature00898

[144] J. An , W. Pickett , Phys. Rev. Lett. 2001, 86, 4366. 11328176 10.1103/PhysRevLett.86.4366

[145] G. Profeta , M. Calandra , F. Mauri , Nat. Phys. 2012, 8, 131. 10.1103/PhysRevLett.108.149701 22540827

[146] M. Xue , G. Chen , H. Yang , Y. Zhu , D. Wang , J. He , T. Cao , J. Am. Chem. Soc. 2012, 134, 6536. 22471507 10.1021/ja3003217

[147] C. Cheng , J. Sun , H. Liu , H. Fu , J. Zhang , X. Chen , S. Meng , 2D Mater. 2017, 4, 025032.

[148] R. C. Xiao , D. F. Shao , W. J. Lu , H. Y. Lv , J. Y. Li , Y. P. Sun , Appl. Phys. Lett. 2016, 109, 122604.

[149] C. Wu , H. Wang , J. Zhang , G. Gou , B. Pan , J. Li , ACS Appl. Mater. Interfaces 2016, 8, 2526. 26732306 10.1021/acsami.5b09949

[150] A. A. Kistanov , Y. Cai , K. Zhou , N. Srikanth , S. V. Dmitriev , Y. W. Zhang , Nanoscale 2018, 10, 1403. 29302656 10.1039/c7nr06537j

[151] L. Kou , Y. Ma , L. Zhou , Z. Sun , Y. Gu , A. Du , S. Smith , C. Chen , Nanoscale 2016, 8, 20111. 27897298 10.1039/c6nr07271b

[152] T. Eknapakul , I. Fongkaew , S. Siriroj , R. Vidyasagar , J. Denlinger , L. Bawden , S.‐K. Mo , P. King , H. Takagi , S. Limpijumnong , W. Meevasana , Phys. Rev. B. 2016, 94, 201121.

[153] M. Feng , J. Zhao , T. Huang , X. Zhu , H. Petek , Acc. Chem. Res. 2011, 44, 360. 21413734 10.1021/ar1001445

[154] G. Csányi , P. Littlewood , A. Nevidomskyy , C. Pickard , B. Simons , Nat. Phys. 2005, 1, 42.

[155] L. Chen , H. Li , A. T. S. Wee , ACS Nano 2009, 3, 3684. 19877598 10.1021/nn900811t

[156] J. Zhao , M. Feng , J. Yang , H. Petek , ACS Nano 2009, 3, 853. 19351148 10.1021/nn800834k

[157] M. Feng , J. Zhao , H. Petek , Science 2008, 320, 359. 18420931 10.1126/science.1155866

[158] A. Yamanaka , S. Okada , Appl. Phys. Express 2014, 7, 125103.

[159] S. Hu , J. Zhao , Y. Jin , J. Yang , H. Petek , J. G. Hou , Nano Lett. 2010, 10, 4830. 21049977 10.1021/nl1023854

[160] V. Silkin , J. Zhao , F. Guinea , E. Chulkov , P. Echenique , H. Petek , Phys. Rev. B 2009, 80. 121408.

[161] L. Kong , L. Liu , L. Chen , Q. Zhong , P. Cheng , H. Li , Z. Zhang , K. Wu , Nanoscale 2019, 11, 15605. 31403639 10.1039/c9nr03792f

[162] Z. Zhang , Y. Yang , G. Gao , B. I. Yakobson , Angew. Chem. 2015, 54, 13135. 10.1002/anie.201505425 26331848

[163] G. Liu , H. Wang , Y. Gao , J. Zhou , H. Wang , Phys. Chem. Chem. Phys. 2017, 19, 2843. 28067931 10.1039/c6cp07367k

[164] Z. Wang , T. Lü , H. Wang , Y. Feng , J. Zheng , Phys. Chem. Chem. Phys. 2016, 18, 31424. 27844074 10.1039/c6cp06164h

[165] T. Tsafack , B. I. Yakobson , Phys. Rev. B. 2016, 93, 165434.

[166] B. Mortazavi , M.‐Q. Le , T. Rabczuk , L. Pereira , Phys. E 2017, 93, 202.

[167] H. Sun , Q. Li , X. G. Wan , Phys. Chem. Chem. Phys. 2016, 18, 14927. 27188523 10.1039/c6cp02029a

[168] D. Rao , L. Zhang , Z. Meng , X. Zhang , Y. Wang , G. Qiao , X. Shen , H. Xia , J. Liu , R. Lu , J. Mater. Chem. A 2017, 5, 2328.

[169] B. Peng , H. Zhang , H. Shao , Y. Xu , R. Zhang , H. Zhu , J. Mater. Chem. C 2016, 4, 3592.

[170] B. Mortazavi , A. Dianat , O. Rahaman , G. Cuniberti , T. Rabczuk , J. Power Sources 2016, 329, 456.

[171] H. R. Jiang , Z. Lu , M. C. Wu , F. Ciucci , T. S. Zhao , Nano Energy 2016, 23, 97.

[172] K. Bhise , S. K. Kashaw , S. Sau , A. K. Iyer , Int. J. Pharm. 2017, 526, 506. 28502895 10.1016/j.ijpharm.2017.04.078 PMC5577003

[173] J. Fang , H. Nakamura , H. Maeda , Adv. Drug Delivery Rev. 2011, 63, 136. 10.1016/j.addr.2010.04.009 20441782

[174] H. Nishino , T. Fujita , A. Yamamoto , T. Fujimori , A. Fujino , S.‐i. Ito , J. Nakamura , H. Hosono , T. Kondo , J. Phys. Chem. C 2017, 121, 10587.

[175] M. Fan , Y. Wen , D. Ye , Z. Jin , P. Zhao , D. Chen , X. Lu , Q. He , Adv. Healthcare Mater. 2019, 8, 1900157. 10.1002/adhm.201900157 30968583

[176] S. K. Das , A. Bedar , A. Kannan , K. Jasuja , Sci. Rep. 2015, 5, 10522. 26041686 10.1038/srep10522 PMC4603704

[177] P. Simon , Y. Gogotsi , Nat. Mater. 2008, 7, 845. 18956000 10.1038/nmat2297

[178] J. Yan , Q. Wang , T. Wei , Z. Fan , Adv. Energy Mater. 2014, 4, 1300816.

[179] C. Liu , F. Li , L. Ma , H. Cheng , Adv. Mater. 2010, 22, E28. 20217798 10.1002/adma.200903328

[180] L. Wang , F. Zhang , C. Lin , ACS Nano 2014, 8, 3724. 24601522 10.1021/nn500386u

[181] M. Lukatskaya , O. Mashtalir , C. Ren , Y. Dall'Agnese , P. Rozier , P. Taberna , M. Naguib , P. Simon , M. Barsoum , Y. Gogotsi , Science 2013, 341, 1502. 24072919 10.1126/science.1241488

[182] C. Wu , X. Lu , L. Peng , K. Xu , X. Peng , J. Huang , G. Yu , Y. Xie , Nat. Commun. 2013, 4, 2431. 24026224 10.1038/ncomms3431

[183] M. Acerce , D. Voiry , M. Chhowalla , Nat. Nanotechnol. 2015, 10, 313. 25799518 10.1038/nnano.2015.40

[184] X. Zhang , J. Hu , Y. Cheng , H. Y. Yang , Y. Yao , S. A. Yang , Nanoscale 2016, 8, 15340. 27502997 10.1039/c6nr04186h

[185] C. Zhan , P. Zhang , S. Dai , D.‐e. Jiang , ACS Energy Lett. 2016, 1, 1241.

[186] X. Lu , M. Yu , G. Wang , T. Zhai , S. Xie , Y. Ling , Y. Tong , Y. Li , Adv. Mater. 2013, 25, 267. 23080535 10.1002/adma.201203410

[187] W. Liu , X. Yan , J. Lang , Q. Xue , J. Mater. Chem. 2011, 21, 13205.

[188] Y. Chen , X. Zhang , D. Zhang , P. Yu , Y. Ma , Carbon 2011, 49, 573.

[189] H. Jiang , Z. Lu , M. Wu , F. Ciucci , T. Zhao , Nano Energy 2016, 23, 97.

[190] N. Jena , R. Barros Neves de Araujo , V. Shukla , R. Ahuja , ACS Appl. Mater. Interfaces 2017, 9, 16148. 28443653 10.1021/acsami.7b01421

[191] Y. Shao , H. Wang , Q. Zhang , Y. Li , J. Mater. Chem. C 2013, 1, 1245.

[192] C. Ataca , A. Ethem , S. Ciraci , Phys. Rev. B. 2009, 79, 041406.

[193] A. Ponrouch , A. R. Goñi , M. R. Palacín , Electrochem. Commun. 2013, 27, 85.

[194] R. Alcántara , M. Jaraba , P. Lavela , J. L. Tirado , Chem. Mater. 2002, 14, 2847.

[195] Y. Liu , N. Zhang , L. Jiao , Z. Tao , J. Chen , Adv. Funct. Mater. 2015, 25, 214.

[196] L. Shi , T. Zhao , A. Xu , J. Xu , Sci. Bull. 2016, 61, 1138.

[197] L. Baggetto , P. Ganesh , C.‐N. Sun , R. A. Meisner , T. A. Zawodzinski , G. M. Veith , J. Mater. Chem. A 2013, 1, 7985.

[198] X. Gonze , J. M. Beuken , R. Caracas , F. Detraux , M. Fuchs , G. M. Rignanese , L. Sindic , M. Verstraete , G. Zerah , F. Jollet , M. Torrent , A. Roy , M. Mikami , P. Ghosez , J. Y. Raty , D. C. Allan , Comput. Mater. Sci. 2002, 25, 478.

[199] H. Pan , X. Lu , X. Yu , Y. Hu , H. Li , X. Yang , L. Chen , Adv. Energy. Mater. 2013, 3, 1186.

[200] M. Mortazavi , J. Deng , V. Shenoy , N. Medhekar , J. Power Sources 2013, 225, 207.

[201] V. Chevrier , G. Ceder , J. Electrochem. Soc. 2011, 158, A1011.

[202] X. Chen , L. Wang , W. Zhang , J. Zhang , Y. Yuan , Int. J. Hydrogen Energy 2017, 42, 20036.

[203] S. Haldar , S. Mukherjee , C. V. Singh , RSC Adv. 2018, 8, 20748. 10.1039/c7ra12512g PMC9080804 35542354

[204] J. Li , H. Zhang , G. Yang , J. Phys. Chem. C 2015, 119, 19681.

[205] X. Tang , Y. Gu , L. Kou , Chem. Phys. Lett. 2018, 695, 211.

[206] J. Wang , Y. Du , L. Sun , Int. J. Hydrogen Energy 2016, 41, 5276.

[207] V. Tozzini , V. Pellegrini , Phys. Chem. Chem. Phys. 2012, 15, 80. 23165421 10.1039/c2cp42538f

[208] T. Liu , Y. Chen , H. Wang , M. Zhang , Y. Lihua , C. Zhang , Materials 2017, 10, 1399.

[209] S. l. Er , G. A. de Wijs , G. Brocks , J. Phys. Chem. C 2009, 113, 18962.

[210] L. Li , H. Zhang , X. Cheng , Comput. Mater. Sci. 2017, 137, 119.

[211] X. Gao , Y. Cui , R. M. Levenson , L. W. K. Chung , S. Nie , Nat. Biotechnol. 2004, 22, 969. 15258594 10.1038/nbt994

[212] I. L. Medintz , H. T. Uyeda , E. R. Goldman , H. Mattoussi , Nat. Mater. 2005, 4, 435. 15928695 10.1038/nmat1390

[213] X. Huang , I. H. El‐Sayed , W. Qian , M. A. El‐Sayed , J. Am. Chem. Soc. 2006, 128, 2115. 16464114 10.1021/ja057254a

[214] P. Beard , Interface Focus 2011, 1, 602. 22866233 10.1098/rsfs.2011.0028 PMC3262268

[215] N. K. Prasad , K. Rathinasamy , D. Panda , D. Bahadur , J. Mater. Chem. 2007, 17, 5042.

[216] B. Jang , J.‐Y. Park , C.‐H. Tung , I.‐H. Kim , Y. Choi , ACS Nano 2011, 5, 1086. 21244012 10.1021/nn102722z

[217] L. Cheng , J. Liu , X. Gu , H. Gong , X. Shi , T. Liu , C. Wang , X. Wang , G. Liu , H. Xing , W. Bu , B. Sun , Z. Liu , Adv. Mater. 2014, 26, 1886. 24375758 10.1002/adma.201304497

[218] W. Tao , N. Kong , X. Y. Ji , Y. P. Zhang , A. Sharma , J. Ouyang , B. W. Qi , J. Q. Wang , N. Xie , C. Kang , H. Zhang , O. C. Farokhzad , J. S. Kim , Chem. Soc. Rev. 2019, 48, 2891. 31120049 10.1039/c8cs00823j

[219] C.‐S. Huang , A. Murat , V. Babar , E. Montes , U. Schwingenschlögl , J. Phys. Chem. C 2018, 122, 14665.

[220] M. Qiu , W. Ren , T. Jeong , M. Won , G. Park , D. Sang , L.‐P. Liu , H. Zhang , J. Kim , Chem. Soc. Rev. 2018, 47, 5588. 29882569 10.1039/c8cs00342d

[221] J. R. Choi , K. W. Yong , J. Choi , A. Nilghaz , Y. Lin , J. Xu , X. Lu , Theranostics 2018, 8, 1005. 29463996 10.7150/thno.22573 PMC5817107

[222] P. Ranjan , J. Lee , P. Kumar , A. Vinu , Adv. Mater. 2020, 32, 2000531. 10.1002/adma.202000531 32666554

[223] D. Li , J. Gao , P. Cheng , J. He , Y. Yin , Y. Hu , L. Chen , Y. Cheng , J. Zhao , Adv. Funct. Mater. 2020, 30, 1904349.

[224] Z. Xie , X. Meng , X. Li , W. Liang , W. Huang , K. Chen , J. Chen , C. Xing , M. Qiu , B. Zhang , G. Nie , N. Xie , X. Yan , Research 2020, 2020, 2624617. 32607497 10.34133/2020/2624617 PMC7312787

[225] C.‐S. Huang , A. Murat , V. Babar , E. Montes , U. Schwingenschlogl , J. Phys. Chem. C 2018, 122, 14665.

[226] V. Shukla , J. Wärnå , N. Jena , A. Grigoriev , R. Ahuja , J. Phys. Chem. C 2017, 121, 26869.

[227] D. Cockcroft , V. Hoeppner , J. Dolovich , Chest 1982, 82, 49. 7083936 10.1378/chest.82.1.49

[228] L. Iii , K. Harris , D. Cugell , R. Patterson , J. Allergy Clin. Immunol. 1993, 92, 29. 8335851 10.1016/0091-6749(93)90033-c

[229] N. Latorre , J. Silvestre , A. Monteagudo , Actas Dermo‐Sifiliogr. 2011, 102, 86. 10.1016/j.ad.2010.09.004 21338980

[230] A. Kootenaei , G. Ansari , Phys. Lett. A 2016, 380, 2664.

[231] L. E. Otterbein , F. H. Bach , J. Alam , M. Soares , H. T. Lu , M. Wysk , R. J. Davis , R. A. Flavell , A. M. K. Choi , Nat. Med. 2000, 6, 422. 10742149 10.1038/74680

[232] F. Inchingolo , M. Tatullo , F. Abenavoli , M. Marrelli , A. Inchingolo , M. Gentile , A. Inchingolo , G. Dipalma , Int. J. Med. Sci. 2010, 7, 378. 21060725 10.7150/ijms.7.378 PMC2974166

[233] M. Tatullo , B. Zavan , F. Genovese , B. Codispoti , I. Makeeva , S. Rengo , L. Fortunato , G. Spagnuolo , Appl. Sci. 2019, 9, 3446.

[234] F. Inchingolo , M. Tatullo , F. Abenavoli , M. Marrelli , A. Inchingolo , A. Palladino , A. Inchingolo , G. Dipalma , Int. J. Med. Sci. 2011, 8, 649. 22135610 10.7150/ijms.8.649 PMC3204433

[235] M. Tatullo , S. Gentile , F. Paduano , L. Santacroce , M. Marrelli , Medicine 2016, 95, e5589. 27930577 10.1097/MD.0000000000005589 PMC5266049

[236] I. Asimov , North American AstroPhysical Observatory Cosmic Search 1981, 9, 5.

[237] F. H. Nielsen , S. L. Meacham , J. Evidence‐Based Complementary Altern. Med. 2011, 16, 169.

[238] Z. Jiang , Y. Ding , T. Zhou , X. Fei , Y. Maobin , Chin. J. Conservative Dent. 2004, 14, 339.

[239] M. A. Soriano‐Ursúa , B. C. Das , J. G. Trujillo‐Ferrara , Expert Opin. Ther. Pat. 2014, 24, 485. 24456081 10.1517/13543776.2014.881472

[240] S. A. Rosenberg , J. C. Yang , N. P. Restifo , Nat. Med. 2004, 10, 909. 15340416 10.1038/nm1100 PMC1435696

[241] F. O. Nestle , S. Alijagic , M. Gilliet , Y. Sun , S. Grabbe , R. Dummer , G. Burg , D. Schadendorf , Nat. Med. 1998, 4, 328. 9500607 10.1038/nm0398-328

[242] E. A. Hirschowitz , J. R. Yannelli , Proc. Am. Thorac. Soc. 2009, 6, 224. 19349492 10.1513/pats.200806-048LC

[243] G. P. Dunn , A. T. Bruce , H. Ikeda , L. J. Old , R. D. Schreiber , Nat. Immunol. 2002, 3, 991. 12407406 10.1038/ni1102-991

[244] D. S. Chen , I. Mellman , Nature 2017, 541, 321. 28102259 10.1038/nature21349

[245] G. T. Gibney , L. M. Weiner , M. B. Atkins , Lancet Oncol. 2016, 17, e542. 27924752 10.1016/S1470-2045(16)30406-5 PMC5702534

[246] H. Yue , W. Wei , Z. Gu , D. Ni , N. Luo , Z. Yang , L. Zhao , J. A. Garate , R. Zhou , Z. Su , G. Ma , Nanoscale 2015, 7, 19949. 26419315 10.1039/c5nr04986e

[247] P. Zhao , Z. Jin , Q. Chen , T. Yang , D. Chen , J. Meng , X. Lu , Z. Gu , Q. He , Nat. Commun. 2018, 9, 4241. 30315173 10.1038/s41467-018-06630-2 PMC6185976

[248] I. Ohsawa , M. Ishikawa , K. Takahashi , M. Watanabe , K. Nishimaki , K. Yamagata , K. Katsura , Y. Katayama , S. Asoh , S. Ohta , Nat. Med. 2007, 13, 688. 17486089 10.1038/nm1577

[249] K. Fukuda , S. Asoh , M. Ishikawa , Y. Yamamoto , I. Ohsawa , S. Ohta , Biochem. Biophys. Res. Commun. 2007, 361, 670. 17673169 10.1016/j.bbrc.2007.07.088

[250] B. M. Buchholz , D. J. Kaczorowski , R. Sugimoto , R. Yang , Y. Wang , T. R. Billiar , K. R. McCurry , A. J. Bauer , A. Nakao , Am. J. Transplant. 2008, 8, 2015. 18727697 10.1111/j.1600-6143.2008.02359.x

[251] M. Dole , F. R. Wilson , W. P. Fife , Science 1975, 190, 152. 1166304 10.1126/science.1166304

[252] T. Aihara , N. Morita , N. Kamitani , H. Kumada , K. Ono , J. Hiratsuka , T. Harada , Appl. Radiat. Isot. 2014, 88, 12. 24799334 10.1016/j.apradiso.2014.04.007

[253] R. Henriksson , J. Capala , A. Michanek , S. A. Lindahl , L. G. Salford , L. Franzen , E. Blomquist , J. E. Westlin , A. T. Bergenheim , Radiother. Oncol. 2008, 88, 183. 18336940 10.1016/j.radonc.2006.04.015

[254] F. B. Rolf , H. S. Albert , G. F. Ralph , M. B. Robert , Cancer 1992, 70, 2995.1451084

[255] R. F. Barth , J. A. Coderre , M. G. H. Vicente , T. E. Blue , Clin. Cancer Res. 2005, 11, 3987. 15930333 10.1158/1078-0432.CCR-05-0035

[256] L. I. Zakharkin , V. A. Ol'shevskaya , R. A. Spryshkova , E. Y. Grigor'eva , V. I. Ryabkova , G. I. Borisov , Pharm. Chem. J. 2000, 34, 301.

[257] W. Chen , J. Ouyang , X. Yi , Y. Xu , C. Niu , W. Zhang , L. Wang , J. Sheng , L. Deng , Y. N. Liu , S. Guo , Adv. Mater. 2018, 30, 1703458. 10.1002/adma.201703458 29194780

[258] S. J. Madsen , C. Christie , S. J. Hong , A. Trinidad , Q. Peng , F. A. Uzal , H. Hirschberg , Lasers Med. Sci. 2015, 30, 1357. 25794592 10.1007/s10103-015-1742-5 PMC4730872

[259] Q. Guo , X. T. Shen , Y. Y. Li , S. Q. Xu , J. Huazhong Univ. Sci. Technol. Med. Sci 2017, 37, 74. 10.1007/s11596-017-1783-z 29058274

[260] J. A. Scholl , A. L. Koh , J. A. Dionne , Nature 2012, 483, 421. 22437611 10.1038/nature10904

[261] H. Wei , E. Wang , Chem. Soc. Rev. 2013, 42, 6060. 23740388 10.1039/c3cs35486e

[262] M. Huo , L. Wang , Y. Chen , J. Shi , Nat. Commun. 2017, 8, 357. 28842577 10.1038/s41467-017-00424-8 PMC5572465

[263] Y. Hu , H. Cheng , X. Zhao , J. Wu , F. Muhammad , S. Lin , J. He , L. Zhou , C. Zhang , Y. Deng , P. Wang , Z. Zhou , S. Nie , H. Wei , ACS Nano 2017, 11, 5558. 28549217 10.1021/acsnano.7b00905

[264] Y. Song , X. Wang , C. Zhao , K. Qu , J. Ren , X. Qu , Chem. Eur. J 2010, 16, 3617. 20191629 10.1002/chem.200902643

[265] Y. Huang , Z. Liu , C. Liu , E. Ju , Y. Zhang , J. Ren , X. Qu , Angew. Chem. 2016, 128, 6758. 10.1002/anie.201600868 27098681

[266] Y. Ji , H. Dong , Y. Li , ChemistrySelect 2017, 2, 10304.

[267] Y. S. Wang , F. Wang , M. Li , B. Xu , Q. Sun , Y. Jia , Appl. Surf. Sci. 2012, 258, 8874.

